# From east to west across the Palearctic: Phylogeography of the invasive lime leaf miner *Phyllonorycter issikii* (Lepidoptera: Gracillariidae) and discovery of a putative new cryptic species in East Asia

**DOI:** 10.1371/journal.pone.0171104

**Published:** 2017-02-10

**Authors:** Natalia Kirichenko, Paolo Triberti, Issei Ohshima, Julien Haran, Bong-Kyu Byun, Houhun Li, Sylvie Augustin, Alain Roques, Carlos Lopez-Vaamonde

**Affiliations:** 1 Sukachev Institute of Forest SB RAS, Federal Research Center «Krasnoyarsk Science Center SB RAS», Krasnoyarsk, Russia; 2 Siberian Federal University, Krasnoyarsk, Russia; 3 INRA, UR0633 Zoologie Forestière, Orléans, France; 4 Museo Civico di Storia Naturale, Verona, Italy; 5 Department of Life and Environmental Sciences, Kyoto Prefectural University, Kyoto, Japan; 6 UMR CBGP (INRA, CIRAD, IRD, SupAgro), Montpellier, France; 7 Department of Biological Science and Biotechnology, Hannam University, Daejeon, South Korea; 8 College of Life Sciences, Nankai University, Tianjin, China; 9 Institut de Recherche sur la Biologie de l’Insecte, CNRS UMR 7261, Université François-Rabelais de Tours, UFR Sciences et Techniques, Tours, France; National Cheng Kung University, TAIWAN

## Abstract

Knowing the phylogeographic structure of invasive species is important for understanding the underlying processes of invasion. The micromoth *Phyllonorycter issikii*, whose larvae damage leaves of lime trees *Tilia* spp., was only known from East Asia. In the last three decades, it has been recorded in most of Europe, Western Russia and Siberia. We used the mitochondrial cytochrome c oxidase subunit I (COI) gene region to compare the genetic variability of *P*. *issikii* populations between these different regions. Additionally, we sequenced two nuclear genes (28S rRNA and Histone 3) and run morphometric analysis of male genitalia to probe for the existence of cryptic species.

The analysis of COI data of 377 insect specimens collected in 16 countries across the Palearctic revealed the presence of two different lineages: *P*. *issikii* and a putative new cryptic *Phyllonorycter* species distributed in the Russian Far East and Japan. In *P*. *issikii*, we identified 31 haplotypes among which 23 were detected in the invaded area (Europe) and 10 were found in its putative native range in East Asia (Russian Far East, Japan, South Korea and China), with only two common haplotypes. The high number of haplotypes found in the invaded area suggest a possible scenario of multiple introductions. One haplotype H1 was dominant (119 individuals, 67.2%), not only throughout its expanding range in Europe and Siberia but, intriguingly, also in 96% of individuals originating from Japan. We detected eight unique haplotypes of *P*. *issikii* in East Asia. Five of them were exclusively found in the Russian Far East representing 95% of individuals from that area. The putative new cryptic *Phyllonorycter* species showed differences from *P*. *issikii* for the three studied genes. However, both species are morphologically undistinguishable. They occur in sympatry on the same host plants in Japan (Sendai) and the Russian Far East (Primorsky krai) without evidence of admixture.

## Introduction

The number of non-native terrestrial arthropods established in Europe has increased exponentially during the second half of the 20th century [1]. Most of these alien species introduced to Europe originate from Asia [1, 2] and can cause significant ecological impact [3]. Many phytophagous insects arrive to Europe with the trade of ornamental plants [4]. Others move westwards as stowaways inadvertently transported with imports of goods from Asia [5], with anthropogenic transportation or expand their geographic range on their own [6].

Micromoths of the family Gracillariidae are an important group of herbivores with several species that are pests of agricultural crops, orchards and ornamental woody plantations worldwide [2, 7]. In recent decades, the damage inflicted by some species of gracillariids has increased remarkably, especially in artificial plantations in cities, parks and gardens. Some species have expanded over large areas from their native regions [8, 9, 10]. Among them, the lime leaf miner *Phyllonorycter issikii* Kumata, 1963, the plane leaf miner *P*. *platani* Staudinger, 1870, the firethorn leaf miner *P*. *leucographella* (Zeller, 1850), the horse-chestnut leaf miner *Cameraria ohridella* Deschka & Dimić, 1986, the locust digitate leaf miner *Parectopa robiniella* Clemens, 1863, *Macrosaccus robiniella* (Clemens, 1859)–all these micromoths have colonized many European countries attacking woody plants of ornamental value [11].

To understand the success of invasive species and establish efficient measures of control, it is important to determine the invasion pathways, sources (populations/regions) of invasion, number of introductions, and the spatial distribution of intraspecific genetic diversity and to assess cryptic diversity, particularly the presence of deeply divergent lineages that are morphologically undistinguishable [12]. However, such information is missing for most invasive species.

Here we address some of these questions using the invasive lime leaf-mining moth *Phyllonorycter issikii* Kumata, 1963 as model system. This species is considered to be native to East Asia (Japan, South Korea and the Russian Far East) [13, 14, 15]. Outside this region, it was first documented in 1985 from *Tilia* plantations in Moscow [16]. During the last three decades, *P*. *issikii* was progressively recorded in Eastern and Western Europe [6, 17, 18]. Presently, the southern border of the insect distribution in Europe runs through Croatia [19], Bulgaria [20] and Serbia [21], reaching Northern Italy [22]. Northward, the moth occupied Finland [23], Baltic countries [24], distributed over Poland [6], Germany [25] and Netherlands [26]. In the west, the insect was detected in north-eastern part of France [27] and in 2011 in eastern part of Belgium [28]. So far, Eastern Belgium (area of Zutendaal) seems to be the current western limit of the moth expansion. Eastward of Moscow, *P*. *issikii* colonized most of Western Russia [17], reached Urals and Western Siberia [29].

In the last decade, high population densities of *P*. *issikii* have been often documented in Western Russia and Western Siberia ([17, 30], Kirichenko pers. obs.). In Eastern Asia, an outbreak of *P*. *issikii* was observed in 2002 in Hokkaido, Japan (Lopez-Vaamonde & Ohshima pers. obs.).

Out of the over 410 species of *Phyllonorycter* described worldwide, only one *P*. *issikii* is specialized to develop on *Tilia* spp. (Malvales: Malvaceae) in the Palearctic. The larvae of *P*. *issikii* mine leaves of lime trees and can cause considerable aesthetical damage in urban parks and gardens. In Western Russia, the damage of mining larvae on lime trees has a negative effect on honey production, since heavily attacked trees produce less flowers than unattacked trees [17, 31]. In addition, in Western Siberia, *P*. *issikii* threatens the conservation of vulnerable Tertiary relic lime stands [32].

During westwards expansion, *P*. *issikii* has switched to *Tilia* species which do not exist in Eastern Asia. In Japan, *P*. *issikii* larvae mine leaves of *Tilia maximowicziana*, *T*. *japonica* and *T*. *kiusiana* [13], whereas in Europe, Western Russia and Siberia, they feed on *T*. *cordata* but also on *T*. *platyphyllos*, *T*. *tomentosa*, *T*. *× euchlora*, *T*. *× europaea*, *T*. *sibirica* [6, 17, 29]. In Siberia (particularly in Barnaul) and in Western Russia (Moscow), *P*. *issikii* also attacks American linden *Tilia americana*, a common plant native to the eastern part of the United States introduced in some Russian botanical gardens [29, 32]. These hosts are not co-involved with *P*. *issikii* and they turned to be susceptible to this novel pest.

Across its modern range, *P*. *issikii* has two generations per year [6, 33]. Leaf mines can be found through June-October. The larvae form blotch mines on underside of leaves. Upper side mines are exceptional but have been recorded in Europe [6] and in Russia (Siberia) in dense populations (Kirichenko pers. obs.). Larvae pupate within leaf mines. The species overwinters as adult [6].

Genetic markers (mitochondrial and nuclear DNA, microsatellites) are commonly used to detect invasive organisms, identify their origin, investigate patterns of historical movement and study the role of genetic variation in invasion success [9, 10, 34, 35, 36]. Some studies have focused on the ecology and biology of invasion of the lime leaf miner [6, 17, 18, 31]. However, nothing is known about its phylogeography and genetics of invasion. Here we studied mitochondrial DNA sequence variation across *P*. *issikii* distribution range from its presumable native regions (East Asia) to its most recently invaded regions (Europe, Western Russian and Siberia). Our aim was to identify the haplotypes which most commonly represent in the invaded region and to investigate they occurrence in East Asia. In addition, we gathered morphometric and nuclear data to test whether the different genetic clusters, revealed by the phylogeographic analysis, represent a complex of cryptic species.

## Materials and methods

### Sample collection

**Fig 1:**
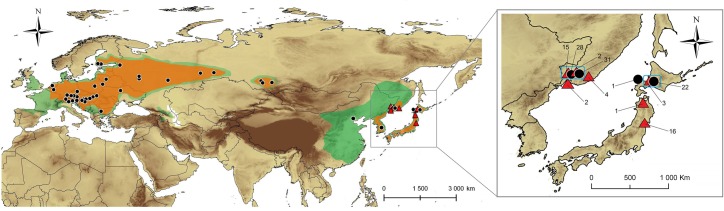
Sampling area in the Palearctic. The present distribution of *Phyllonorycter issikii* is shaded in orange and the distribution of its host plant *Tilia*–in green. Localities where specimens of *Phyllonorycter issikii* and putative new *Phyllonorycter* species were collected are marked by black circles and red triangles respectively (details about localities are given in S1 Table). The insertion illustrates the close up of sampling of *P*. *issikii* (black circles) and *Phyllonorycter* sp. n. (red triangles) in East Asia and number of specimens per location; blue squares show the contact zones where both species occur together (two locations in the Russian Far East–Mountain Taiga Station and Observatory, and one location in Japan–Sapporo).

Overall, 387 individual insects were collected in 16 countries and 65 localities across present range of *Phyllonorycter issikii* in the Palearctic region (Fig 1). These localities are plotted on the map showing distribution of *P*. *issikii* and its host plant *Tilia* spp. extracted from various sources [6, 13, 14, 15, 17, 21, 25, 28, 29, 37, 38, 39, 40, 41, 42]. The coordinates of localities are provided in S1 Table.

Altogether, 256 specimens were sampled from the presumable invaded area: from 12 European countries and Russia (western part of the country and Siberia). The rest 131 specimens were collected in the presumable native range in Eastern Asia (Russian Far East, Japan, South Korea and China) (Fig 1; S1 Table).

In total, 346 (89%) specimens out of 387 studied specimens were collected directly from their mines at larval and pupal stages, 149 individuals were reared to adults. Thus, host plants of all those specimens were determined. For 41 adults collected in the field by sweeping host plants remained unknown. All larvae and pupae and most adults were stored in 96% ethanol solution and kept at -20°C until DNA extraction. Overall, 72 adults were pinned and stored for the present study.

We studied *P*. *issikii* samples from 10 different *Tilia* host plant species, six species in East Asia (*T*. *mandshurica*, *T*. *maximowicziana*, *T*. *amurensis*, *T*. *japonica*, *T*. *taquetii* and *T*. *mongolica*) and four species in Europe, Western Russia and Siberia (*T*. *cordata*, *T*. *sibirica*, *T*. *platyphyllos* and *T*. *dasystyla)*; 13 lime trees surveyed in the Russian Far East and in Europe have not been identified to the species level. In total, 155 out of 387 studied specimens (i.e. 40%) were collected from *T*. *cordata* (S1 Table). Detailed collection data and host plant list is provided in S1 Table. In order to avoid sampling the related individuals, whenever possible, a single individual insect was collected per tree, and up to 30 trees were sampled by locations.

As no other monophagous species of *Phyllonorycter* exists on *Tilia* in the Palearctic, we used the related Nearctic species, *Phyllonorycter lucetiella* (Clemens, 1859), developing on *Tilia*, to root the genetic trees. One pupa of *P*. *lucetiella* was collected on *T*. *americana* in Quebec (Canada).

All specimens were collected legally in public parks and gardens and governmental forest, where no special permission was required. Collection of *P*. *issikii* specimens in the protected Siberian lime groove in Kuzedeevo (Kemerovskaya oblast, Russia) was done as part of a long-term monitoring program.

### DNA sequence analysis

Altogether, 377 out of 387 specimens from 16 countries and 65 locations were analyzed with mitochondrial gene cytochrome c oxidase subunit I (COI) (Table 1).

**Table 1:** Number of DNA-barcoded specimens of *Tilia*-feeding *Phyllonorycter* species from different countries.

№	Country	Number of localities	Number of specimens^1^	Host plant^2^
**Presumable invasive range**				
1	Austria	14	30	*T*. *platyphyllos* (7); *T*. *cordata* (4); X(19)
2	Bulgaria	1	22	*T*. *cordata* (20); *Tilia* sp. (2)
3	Czech Republic	1	1	*T*. *platyphyllos* (1)
4	Finland	3	17	*T*. *cordata* (13); X (4)
5	Germany	4	4	UH (4)
6	Hungary	11	21	*T*. *cordata* (17); *T*. *platyphyllos* (4)
7	Italy	2	2	UH (2)
8	Lithuania	1	4	*T*. *platyphyllos* (3); X (1)
9	Netherlands	2	2	*T*. *cordata* (1); *Tilia* sp. (1)
10	Poland	2	4	*Tilia* sp. (4)
11	Russia (western part, the Urals, Siberia)	11	120	*T*. *amurensis* (9); *T*. *cordata* (83); *T*. *dasystyla* (11); *T*. *sibirica* (17)
12	Slovenia	2	6	*T*. *cordata* (4); *Tilia* sp. (1); X (1)
13	Ukraine	1	13	*T*. *cordata* (13)
**Presumable native range**				
14	Japan	4	23+20	*T*. *maximowicziana* (22+3); *T*. *japonica* (1+16); *Tilia* sp. (1)
15	Russia (Russian Far East)	4	59+23	*T*. *amurensis* (3+7); *T*. *mandshurica* (49+7); *T*. *taquetii* (7+5); *Tilia* sp. (4*)*
16	South Korea	1	5	*T*. *mandshurica* (5)
17	China	1	1	*T*. *mongolica* (1)
**Total**		**65**	**377**	*T*. *cordata* **(155)**; *T*. *mandshurica* **(61)**; *T*. *maximowicziana* **(25)**; *T*. *amurensis* **(19)**; *T*. *japonica* **(17)**; *T*. *sibirica* **(17)**; *T*. *platyphyllos* **(15)**; *Tilia* sp. **(13)**; *T*. *taquetii* **(12)**; *T*. *dasystyla* **(11)**; *T*. *mongolica* **(1)**; X **(31)**

^**1**^**Number of specimens:** in the presumable native range, the specimens were represented by *Phyllonorycter issikii* and by the putative new species, which was found on the same host plants in the same localities. Number of specimens of the putative new species is underlined.

^2^**Host plant** is known only for specimens collected directly from mines; host is unknown (X) for adult moths freely collected in field.

For 285 specimens, DNA extraction, PCR amplification and sequencing of the barcode fragment were done at the Laboratory of Forest Zoology URZF, INRA (Orléans, France). DNA extracts were prepared from larvae, pupae, hind legs or abdomens of pinned adults using NucleoSpin tissue XS kit, Macherey-Nagel, Germany according to the manufacturer’s protocol. The COI barcoding fragment, 658 bp, was amplified via PCR using the primers LCO (5' GGT CAA CAA ATC ATA AAG ATA TTG G 3') and HCO (5' TAA ACT TCA GGG TGA CCA AAA AAT CA 3') and following standard conditions for the reaction [43]. PCR products were purified using the QIAGEN QIAquick PCR Purification Kit. Sequence reaction was done by the Sanger method with ABI Prism Big Dye Terminator 3.1cycle sequencing kit (25 cycles of 10 s at 96°C, 5 s at 50°C, 4 min at 60°C).

For 47 specimens, the same COI fragment was amplified at the Canadian Centre for DNA Barcoding (CCDB–Biodiversity Institute of Ontario, University of Guelph) using a slightly different sequencing set C_LepFolF/C_LepFolR, following the standard high-throughput protocol [44]. Furthermore, 45 *P*. *issikii* specimens from Europe, barcoded with LCO and HCO primers were provided by our colleagues for analysis (S1 Table).

In addition, 49 previously DNA-barcoded specimens from presumable native and invaded areas were amplified for two nuclear fragments: histone 3 (H3) using primers H3F (5’ ATG GCT CGT ACC AAG CAG ACG GC) and H3R (5’ ATA TCC TTG GGC ATG ATG GTG AC) (Colgan et al 1998) and 28S rDNA (28S) using primers D1F (5' ACC CGC TGA ATT TAA GCA TAT) and D3R (5’ TAG TTC ACC ATCTTT CGG GTC [45]. These two nuclear genes are widely used in reconstruction of the phylogeny of Gracillariidae [46, 47, 48] and were used here to assess cryptic diversity. PCR amplification with H3 gene was done at the following conditions: 40 cycles (1 min at 94°C, 1 min at 45°C, 1 min at 65°C) and with 28S 30 cycles (45 s at 94°C, 50 s at 57°C, 1 min at 72°C) [45].

One specimen of the North American *Tilia*-feeding *Phyllonorycter lucetiella* was also sequenced for COI, Histone 3 and 28S genes to be used as outgroup.

All specimens were bidirectionally sequenced using BigDye sequencing protocol (Applied Biosystems 3730xl). All electropherograms (for mitochondrial and nuclear genes) were checked manually in CodonCode Aligner ver. 3.7.1. (CodonCode Corporation) to assess their quality. Alignment was done in BIOEDIT ver. 7.1.7 and each nucleotide variation was double checked to confirm each haplotype [49]. No pseudogenes or stop codons were detected in the sequences. All COI sequences of *P*. *issikii* and the putative new species were trimmed to the same length for a final alignment (618bp). Nuclear gene sequences for both insects represented the whole length, i.e. 328 bp for H3 and 940 bp for 28S.

Details on the collecting data for each specimen, as well as a photograph of vouchers, sequence records, trace files, and primer sequences used for PCR amplification, together with GenBank accession numbers are available through the following dataset (dx.doi.org/10.5883/DS-TILIAPHY) in BOLD (www.boldsystems.org).

### Morphology

**Fig 2:**
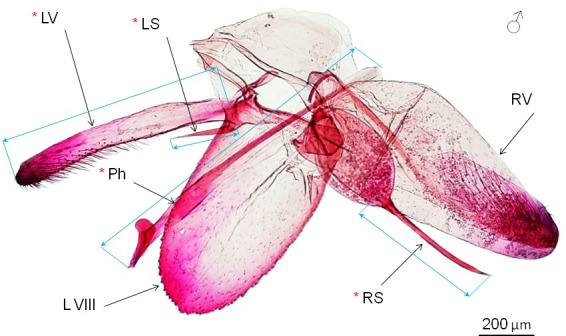
Male genitalia of *Phyllonorycter issikii* (Russia: Russian Far East, Ussuriysk, Gornotayejnoe, forest near astrophysical observatory, *Tilia mandshurica*, 17.VII.2013, adult reared from mine, genitalia slide ID: 3975male; DNA barcode ID: ISSIK174-14). Measured characters (indicated by red asterisk): left valva (LV), spine of the left basal process i.e. left spine (LS), spine of the right basal process i.e. right spine (RS), phallus (Ph). Measured length of each character is indicated by blue lines. Right valva (RV) and VIII abdominal sternite (LVIII) were not used for measurements (see Materials and Methods).

External morphology (wing pattern) has been examined in 72 dried, pinned and mostly set specimens (62 out of 72 specimens were DNA-barcoded) (S1 Table). In addition, we studied male genitalia of 56 moths (46 of these moths were DNA-barcoded) (S1 Table). Morphometric analysis was carried out on genital preparations of 33 males from the presumable invaded area (Bulgaria, Finland, Hungary, Italy, Poland, Western Russia and Siberia) and of 21 males from the presumable native region (Russian Far East, Japan, South Korea). Genitalia dissections and slide mounts followed by the standard technique [50]. Length of the four genital parameters: phallus, left valva, and spines of left and right basal processes (hereinafter–left spine and right spines) was measured on a Leica M 165C stereomicroscope and expressed in μm (Fig 2). Male genitalia of *P*. *issikii* is asymmetric, with a big right valva (Fig 2), which was impossible to measure precisely due to its deformation when arranging slides with genital structures. Thus it was not included into analysis.

### Statistical analysis

Sampling area and geographic distribution of COI haplotypes was displayed with an altitudinal background using ArcGIS 9.3 [51]. For European countries, data were pooled together to represent a particular country, whereas in Russia, given the large size of the country, and in the Eastern Asia, haplotype diversity was indicated for all studied localities.

Haplotype diversity was calculated using DnaSP v 5.10.1 [52]. Intra- and interspecific genetic distances were estimated using the Kimura 2-parameter model implemented and a barcode gap analysis was carried out within the analytical tools available in BOLD. A neighbor-joining (NJ) tree was constructed using MEGA 6.0 [53]. Correlation between genetic diversity and number of specimens per location was assessed with nonparametric Spearman rank R (*p* < 0.05). Nonparametric Mann-Whitney U test was used to check for differences in genetic diversity between groups of populations (invaded vs. native areas) because the original data was not normally distributed (Shapiro-Wilk W = 0.27, *p* < 0.0001; Kolmogorov-Smirnov D = 0.46, *p* < 0.01). The analysis was run in STATISTICA 8.0 (Stat Soft. Inc., USA).

To investigate relationship among haplotypes within *P*. *issikii* and a putative new species of *Phyllonorycter*, the median-joining haplotype networks were built with the program TCS 1.21 implementing a statistical parsimony algorithm [54]. To identify sources of genetic variance in the COI sequence data, a nested analysis of molecular variance (AMOVA) was applied using the program Arlequin 3.5.13 [55]. Two variants of grouping by geographical region have been tested. In the first case, two groups were defined: (i) the presumable invaded area: Europe, Western Russia, Siberia and (ii) the presumable native area: Russian Far East, Japan, South Korea and China. In the second case, we tested three groups: the first group (the presumable invaded area) remained as above, whereas the second group (the presumable native area) was divided into two: one subgroup represented the Russian Far East, South Korea and China and the other–Japan, from where the insect was originally described. Three sources of variation were taken into account: among groups, among populations within groups and within populations. The significance of the variance components was assessed with the nonparametric permutation method using 10 000 permutations.

Dataset of male genital measurements, prior to analysis, was tested for normality by the Shapiro-Wilk and Kolmogorov-Smirnov tests [56]. Since morphometric data was normally distributed based on fit distribution procedure for each morphometric parameter (length of phallus: Shapiro-Wilk W = 0.97, *p* = 0.48, Kolmogorov-Smirnov D = 0.06, *p* > 0.20; left valvа: W = 0.96, *p* = 0.11, D = 0.88, *p* > 0.20; left spine: W = 0.93, *p* = 0.12, D = 0.83, *p* > 0.20; right spine: W = 0.91, *p* = 0.15; D = 0.13, *p* > 0.20), the dataset was processed using a linear discriminant analysis [57], with species as categorical dependent variable and four genital characters (length of phallus, left valva, left spine and right spine) as continuous independent variables. Prior to the analysis, 10-fold cross-validation procedure was performed (the averaged Wilks’ λ = 0.26 ± 0.02, F-value = 29.16 ± 2.69, *p* < 0.0001; APCC for the training samples = 98.43 ± 0.54%, APCC for validation samples = 98.33 ± 1.83%), which proved high accuracy of the observed classification [57]. The analyses were performed in STATISTICA 8.0 (Stat Soft. Inc., USA).

## Results

### Molecular systematics of *Phyllonorycter issikii*

**Fig 3:**
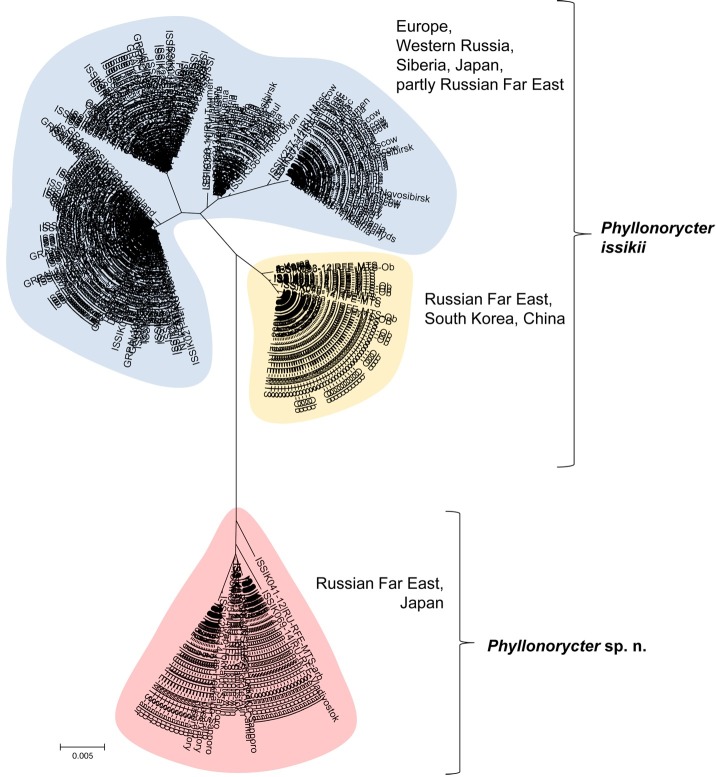
Neighbor-joining COI tree of *Phyllonorycter issikii* and the putative new cryptic species. The two clusters highlighted in blue and yellow correspond to *P*. *issikii* (BOLD:AAC9940), with minimum distance to nearest neighbour of 3.66%. The red cluster corresponds to a putative new cryptic species. The sampled regions are indicated near each cluster (RFE–Russian Far East). The putative new species sympatrically occurs with *P*. *issikii* in the Russian Far East and Japan, sharing the same host plants.

The analysis of 377 DNA barcodes of *Tilia* leaf miners collected in the Palearctic revealed a deep split with two clades: one formed by 334 specimens belonging to *P*. *issikii* and a second clade formed by 43 specimens which could represent an undescribed species from the genus *Phyllonorycter* (Fig 3). There was a barcode gap between the maximum intraspecific divergence (2.96% for *P*. *issikii* and 1.38% for the putative new species) and the minimum distance to the nearest neighbor (3.66%), with average distance (5.13±0.003%) between the two clades (S1 Fig). The two mitochondrial clades were separated by 20 diagnostic mutation steps.

The cluster of the putative new species (shaded in red, Fig 3) was solely represented by specimens from the Russian Far East and Japan. The *P*. *issikii* cluster contained two subgroups of haplotypes: the biggest subgroup, highlighted in blue, included specimens from the presumable invaded area, Japan and the Russian Far East (Fig 3). The smaller subgroup highlighted in yellow was formed by the majority of specimens from the Russian Far East and all specimens from South Korea and China (Fig 3).

**Fig 4:**
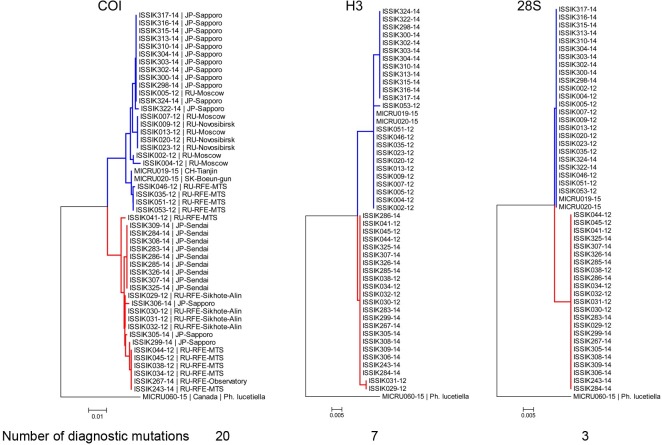
Neighbor-joining COI, Histone H3 and 28S trees showing divergence of *P*. *issikii* and the putative new *Phyllonorycter* species. Same individuals (26 specimens of *P*. *issikii* and 23 specimens of *Phyllonorycter* sp. n.) were sequenced for the three genes: COI, histone 3 H3 and 28S rDNA. The blue clade corresponds to *P*. *issikii*, the red clade corresponds to the putative new species. All trees are rooted with congruent sequences of Nearctic *Tilia*-feeding *Phyllonorycter lucetiella* (Canada, Quebec). Geographical locations are indicated on the NJ COI tree (RU–Russia, RFE–Russian Far East, JP–Japan; SK–South Korea, CH- China).

Forty-nine randomly selected, previously barcoded specimens (26 specimens of *P*. *issikii* from the west and east from both blue and yellow clusters and 23 of the putative new species) were sequenced with two nuclear genes. Both H3 and 28S unequivocally delimited two distinct species with three and seven diagnostic nucleotide substitutions respectively (Fig 4).

The putative new cryptic species was found exclusively in East Asia, which is known as native range of *P*. *issikii*. These insects shared the same host plants (and often the same individual trees): *T*. *amurensis*, *T*. *mandshurica*, *T*. *taquetii* in the Russian Far East and *T*. *japonica* and *T*. *maximowicziana* in Japan (Table 1).

In the Russian Far East, the haplotypes of putative new cryptic species were found in 28.1% of specimens (i.e. 23 specimens out of 82 collected insects were assigned to this putative new species), whereas in Japan the ratio was higher reaching 46.5% (i.e. 20 specimens of the new candidate species out of 43 collected insects) (Fig 1, see the insertion). Despite the putative new cryptic species was less abundant than *P*. *issikii*, it was present in more localities in East Asia than *P*. *issikii*. It was found in four sites in the Russian Far East–(1) in the arboretum of the Mountain-taiga station (MTS), (2) around astrophysical observatory (both sites near Gornotayejnoe, Ussuriysk), (3) around the national park “Call of the Tiger” in the Sikhote-Alin mountains and (4) in the park of Akademgorodok (Vladivostok) (vs. two sites with *P*. *issikii*, i.e. № 1 and 2 mentioned above) and in three sites in Japan–around the cities (1) Sendai, (2) Kuroishi and (3) Sapporo (vs. one site with *P*. *issikii*, i.e. № 3) (Fig 1, see the insertion). The coordinates of the places are given in S1 Table. Thus, the contact zone of *P*. *issikii* and the putative new was found in both the Russian Far East (sites № 1, 2) and Japan (site № 3). In the Russian Far East, the new candidate species occurred both in wild (sites № 2, 3) and in the artificial plantations (sites № 1, 4). In Japan, the putative new species was also found in wild and in artificial plantations of lime trees around Sendai, Kuroishi and Sapporo.

### Phylogeography of *Phyllonorycter issikii*

Overall, 334 individuals *P*. *issikii* were included in the phylogeographic analysis. Among them, 246 individuals were collected in the invaded region (Europe and Russia) and 88 individuals collected in the native region in East Asia (59 in the Russian Far East, 23 in Japan, five in South Korea and one in China).

**Fig 5:**
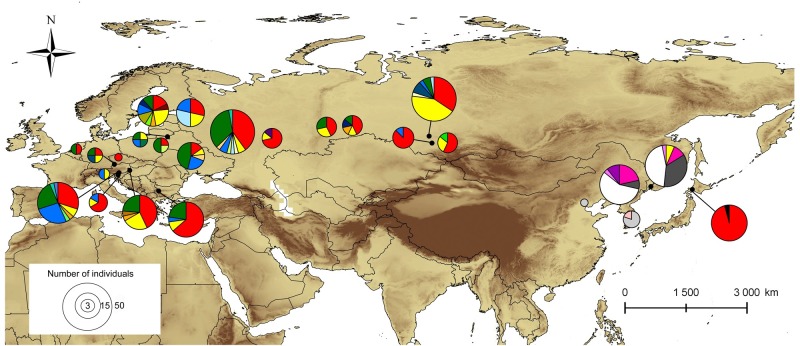
Geographical distribution of the 31 haplotypes of *Phyllonorycter issikii* across its present range in the Palearctic. Each pie chart represents a country except Russia, where each location is indicated. Colors of pie charts refer to particular haplotypes, corresponding to those of the network in Fig 6. Complementary, presence of *P*. *issikii* haplotypes in different countries is provided in S2 Table.

**Fig 6:**
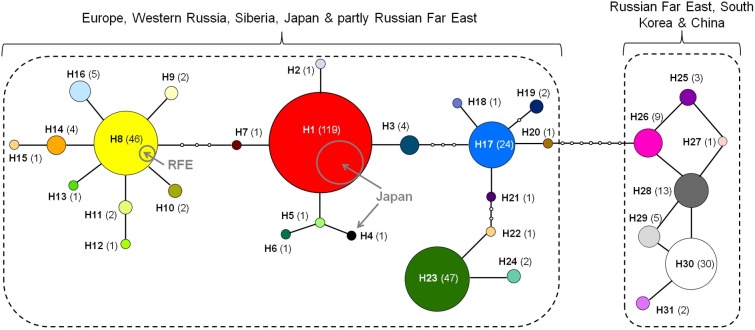
Haplotype network of *Phyllonorycter issikii* in the Palearctic. Different colors correspond to the haplotypes H1-H31. Number of individuals per haplotype is indicated in parentheses. Haplotypes are connected with a 95% confidence level. Each line connecting circles represents a single mutational change. Empty circles indicate intermediate, missing haplotypes. Dotted rectangles indicate the two main geographical structures. Gray circles inserted into the haplotype H1 and H8 show contribution of populations from East Asia. The geographical distribution of all haplotypes is illustrated in Fig 5 and reported in S2 Table.

A total of 31 mitochondrial haplotypes were identified in *P*. *issikii* populations (Figs 5 and 6), with 23 haplotypes detected in the presumable invaded area and ten haplotypes discovered in East Asia (Fig 5). Out of those ten haplotypes, eight were unique, five haplotypes being found only in the Russian Far East, one in Japan and two in South Korea and China (Fig 5).

Haplotype distribution in the studied countries is indicated in Table 2. The highest genetic diversity (nine haplotypes per location) was found in two countries: Finland and Russia (Moscow region) (Table 2). Overall, haplotype diversity was significantly correlated to sampling effort (number of individuals sampled per population) (Spearman correlation, rS = 0.76; N = 25; *p* = 0.00001).

**Table 2:** Haplotypes of *Phyllonorycter issikii* found across its present range in the Palearctic.

№	Country, locality^1^	Haplotype and specimen number (in brackets)	Total number of haplotypes	Total number of specimens
**Presumable invasive range**				
1	Austria	H1(9), H5(1), H8(2), H14(1), H17(8), 18(1), H23(7), H24(1)	8	30
2	Bulgaria	H1(14), H8(2), H17(1), H23(5)	4	22
3	Czech Republic	H1(1)	1	1
4	Finland	H1(3), H7(1), H8(4), H10(2), H12(1), H14(1), H17(2), H19(1), H23(2)	9	17
5	Germany	H1(1), H3(1), H15(1), H26(1)	4	4
6	Hungary	H1(9), H8(5), H14(1), H20(1), H23(5)	5	21
7	Italy	H8(1), H17(1)	2	2
8	Lithuania	H6(1), H8(1), H17(1), H23(1)	4	4
9	Netherlands	H1(1), H23(1)	2	2
10	Poland	H1(1), H11(1), H23(2)	3	4
11	Slovenia	H1(4), H8(1), H17(1)	3	6
12	Ukraine	H1(2), H8(1), H15(1), H17(3), H23(6)	5	13
13	Russia (Mos)	H1(14), H8(2), H9(1), H11(1), H16(1), H17(2), H22(1), H23(12), H24(1)	9	35
14	Russia (StPb)	H1(4), H8(3), H16(4), H17(3)	4	14
15	Russia (Uly)	H1(5), H8(1), H21(1)	3	7
16	Russia (Yek)	H1(3), H8(2), H23(2)	3	7
17	Russia (Nov)	H1(12), H2(1), H3(3), H8(15), H9(1), H17(1), H23(2)	7	35
18	Russia (Bar)	H1(7), H17(1)	2	8
19	Russia (Kuz)	H1(4), H8(2), H13(1)	3	7
20	Russia (Tyu)	H1(3), H8(1), H14(1), H19(1), H23(1)	5	7
**Presumable native range**				
21	Russia, Rus Far East (MTS)	H25(3), H26(6), H28(2), H30(16), H31(1)	5	28
22	Russia, Rus Far East (Obs)	H8(2), H26(3), H28(11), H30(14), H31(1)	5	31
23	Japan (Sapporo)	H1(22), H4(1)	2	23
24	South Korea (Boeun-gun)	H27(1), H29(4)	2	5
25	China (Tianjin)	H29(1)	1	1

^**1**^ **Country, locality:** localities are listed only for Russia, Japan, South Korea and China.

Haplotype and nucleotide diversity of *P*. *issikii* from the presumable invaded area (Europe, Western Russia and Siberia) and native area (East Asia) are given in Table 3. In the presumable invaded area, we found 23 haplotypes for 246 specimens, with median number of haplotypes per individual 0.013 **±** 0.011. In the presumable native area we revealed 10 haplotypes for 88 specimens, with median number of haplotypes per individual 0.019 **±** 0.013. This difference was not significant (Mann-Whitney U test: Z = -0.84, *p* = 0.397).

**Table 3:** Haplotype and nucleotide diversity of *P*. *issikii* in the presumable invaded and native areas in the Palearctic.

Range^1^	№ speci-mens	№ coun-tries	№ locali-ties	№ haplo-types	Distribution of haplotypes H1-H29 (number of specimens with a certain haplotype)	Haplotype diversity (value ± StDiv)	Nucleotide diversity (value ± StDiv)
**Presumable invasive**	246	13	55	23	H1(97), H2(1), H3(4), H5(1), H6(1), H7(1), H8(44), H9(2), H10(1), H11(2), H12(1), H13(1), H14(4), H15(1), H16(5), H17(24), H18(1), H19(2), H20(1),H21(1), H22(1), H23(47), H24(2)	0.768±0.018	0.0085±0.0061
**Presumable native**	88	4	10	10	H1(22), H4(1), H8(2), H25(3), H26(9), H27(1), H28(13), H29 (5), H30(30), H31(2)	0.816±0.022	0.0239±0.0132

^**1**^**Range: the presumable invasive range:** Europe, Western Russia, Siberia; **the presumable native range:** East Asia–the Russian Far East, Japan, South Korea and China.

The parsimony-based haplotype network showed two main clusters separated by seven mutational steps (Fig 6). The larger cluster contained in total 271 specimens, i.e. all specimens from Europe, Western Russia, Siberia and Japan and three samples from the Russian Far East. The second cluster was represented by 63 individuals, i.e. by 57 specimens from the Russian Far East, five from South Korea and one from China.

The larger cluster was formed by the dominant haplotype H1, shaded in red (40% of specimens from the presumable invaded area) and by the three subdominant haplotypes H8, yellow (19%), H17, blue (17%) and H23, green (10%). These haplotypes centred star-like clusters, which however had no clear geographical structure (Fig 6). These clusters were linked with two to three mutation steps. Only two haplotypes H1 (red) and H8 (yellow) were common for the west and the east. In East Asia, H1 was present in Japan but not in the Russian Far East, whereas H8 in the latter but not in Japan (Table 2, Fig 5). Haplotype H1 was most abundant in the Palearctic. In the invaded area, it was found in 10 out of 12 European countries and in all examined locations in Russia (Western part and Siberia), with about 40% of specimens carrying this haplotype. In East Asia, the haplotype H1 was exclusively found in Japan, with 96% of individuals. Curiously, the unique haplotype H4, found only in one Japanese specimen, was linked to the dominant haplotype H1 through two mutational steps.

The second cluster contained seven unique haplotypes, five of them were found only in the Russian Far East and other two in South Korea and China (Fig 6). No one haplotype was shared between these locations, except for H29, which was shared between Korea and China. The most abundant haplotype was H30 (white), with 51% of the specimens in this cluster, followed by the haplotype H28 (gray), with 22% of specimens from the Russian Far East (Fig 5).

In the haplotype network, overall 16 haplotypes were missing. Thirteen missing haplotypes linked the most distant subdominant haplotype H8 in Europe with the Russian Far East cluster through H7, H1, H3, H17 and H20. The rest three missing haplotypes were situated around the subdominant haplotype H17 in Europe. Twelve haplotypes (H2, H4-H7, H12, H13, H15, H18, H20-H22) were each represented by a single individual on the west.

Analysis of molecular variance revealed significance of all the considered sources of variations (Table 4). Variation among groups was the highest (25.82%) when three geographical groups were analyzed: (i) the presumable invaded area (Europe, Western Russia, Siberia) and the presumable native range–(ii) Russian Far East, South Korea and China, and (iii) Japan (Table 4, see variant № 2). When East Asia was treated as one group and analyzed against the group from the west, the difference among groups decreased but remained statistically significant due to high number of unique haplotypes found in the east, particularly in the Russian Far East. The greatest amount of total nucleotide variation was accounted by differences among individuals within populations: 68.82% when three geographical groups were analyzed (the presumable invaded area, Russian Far East, Japan) and 75.27% when two groups were analyzed (the presumable invaded and native areas) (Table 4).

**Table 4:** Analysis of molecular variance (AMOVA) for populations of *Phyllonorycter issikii* in the Palearctic based on mitochondrial gene COI.

Source of variation*	Variance, %	Ф-statistics	*p*-value
**Grouping by geographical region, variant № 1**			
Among groups (Ф_CT_)	11.25	0.11250	<0.05
Among populations within groups (Ф_SC_)	13.48	0.15186	<0.001
Within population (Ф_ST_)	75.27	0.24728	<0.001
**Grouping by geographical region, variant № 2**			
Among groups (Ф_CT_)	25.82	0.25819	<0.001
Among populations within groups (Ф_SC_)	5.36	0.07222	<0.001
Within population (Ф_ST_)	68.82	0.31176	<0.001

* **Source of variation**:

**Grouping by geographical region, variant № 1:** two groups involved–(i) presumable invaded area (Europe, Western Russia, Siberia) and (ii) presumable native area (Russian Far East, Japan, South Korea and China).

**Grouping by geographical region, variant № 2:** three groups involved–(i) presumable invaded area (same as above), (ii) Japan, (iii) Russian Far East, South Korea and China.

*Phyllonorycter issikii* is a new record for China. We found a few empty mines and a pupa of *P*. *issikii* on the north-east of the country (Baxian mountain nature reserve, Tianjin, coordinates: 40°09’N, 117°43’E) in July 2015. For the first time, we also documented *P*. *issikii* on the new host plants: on *Tilia mongolica* in China (same location as mentioned above), *T*. *taquetii* in the Russian Far East (arboretum of the Mountain-taiga station, Gornotayejnoe, Ussuriysk), and *T*. *dasystyla* in Western Russia (Moscow).

### Phylogeography of the putative new cryptic species

**Fig 7:**
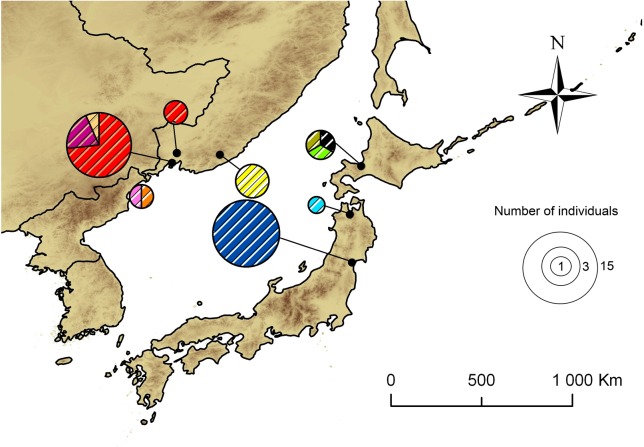
Geographical distribution of the 11 haplotypes of the putative new *Phyllonorycter* species in East Asia. Each pie chart represents one locality in the Russian Far East and Japan. Each color corresponds to a particular haplotype, referring to those of the network on Fig 8. The total surface of each pie chart is proportional to sample size indicated in the legend.

In our study, 43 specimens, originally collected as *P*. *issikii*, represented the putative cryptic species of *Phyllonorycter*, with 11 haplotypes distributed across the Russian Far East and Japan (Fig 7). None of these haplotypes was common for the Russian Far East and Japan. In the Russian Far East, the haplotype A was most abundant, with 30.2% specimens collected throughout East Asia (Fig 7). In Japan, the most dominant was the haplotype K, with 37.2% specimens collected in East Asia. In total, seven out of 11 haplotypes were represented by one individual (i.e. the haplotypes B, D, E, F, H, I and J) in the Russian Far East and Japan.

**Fig 8:**
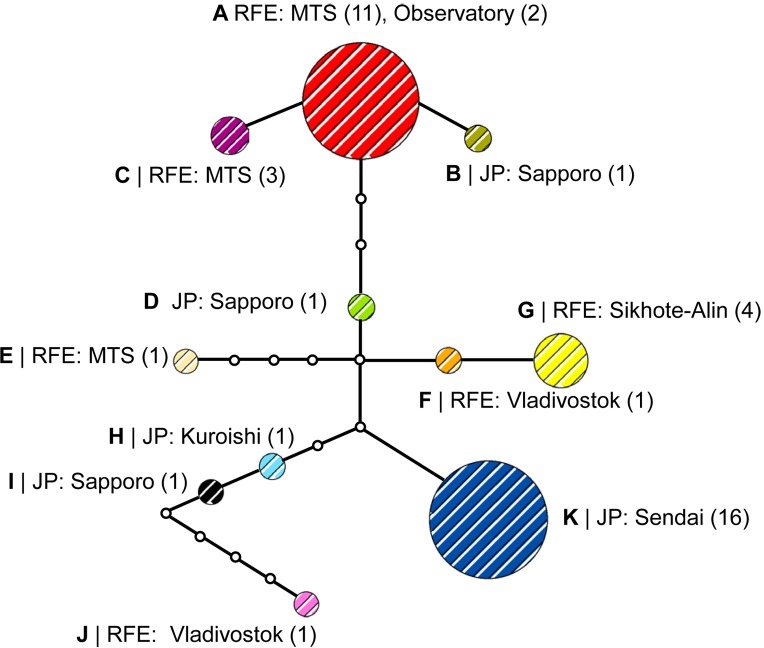
Haplotype network of the putative new *Phyllonorycter* species in East Asia. Different colors correspond to the haplotypes A-K. Number of individuals per haplotype in each locality is indicated in parentheses. Haplotypes are connected with a 95% confidence limit. Each line in the network represents a single mutational step. Empty circles indicate intermediate, missing haplotypes. Abbreviation: RFE–Russian Far East, MTS–Mountain taiga station (Gornotayejnoe, Ussuriysk), JP–Japan.

The parsimony-based haplotype network showed a branched pattern, with two main haplotypes A and K, separated by five mutations (Fig 8). Despite the haplotypes were specific for the Russian Far East and Japan, the haplotype network had no clear geographical structure. The haplotypes from the Russian Far East and Japan were found without particular order within the haplotype network. For example, the haplotype B, represented by one individual from Sapporo (Japan), radiated directly from the dominant haplotype A found exclusively on the Russian Far East, whereas the haplotype F from Vladivostok (Russia) was linked with the main Japanese haplotype K via only two mutational steps (Fig 8). In the haplotype network, 12 haplotypes were missing; some of them could probably be common for both geographical regions.

### Morphometric analysis

The study of moth wing pattern showed no differences between western and eastern populations of *P*. *issikii* neither between *P*. *issikii* and the cryptic *Phyllonorycter* species from East Asia. Nevertheless, morphometric analysis of male genitalia pointed at some differences between geographically remote *P*. *issikii* populations.

**Fig 9:**
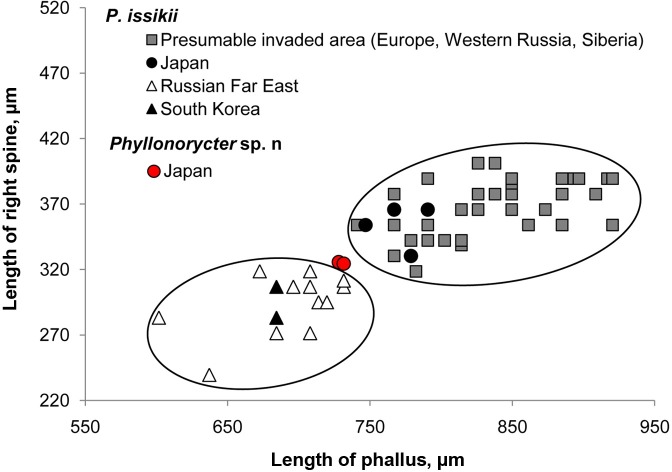
Spatial distribution of length of phallus and right spine in *Phyllonorycter issikii* and the putative new *Phyllonorycter* species. Specimens of *P*. *issikii* originating from Japan and the presumable invaded area (Europe, Western Russia, Siberia) were pooled together due to absence of significant difference between countries or localities in the data set (Wilks’ λ = 0.47, *p* = 0.28, N = 38). The same procedure was done for the specimens of *P*. *issikii* from the Russian Far East and South Korea (Wilks’ λ = 0.55, *p* = 0.21, N = 14). Two specimens of the putative new cryptic species of *Phyllonorycter* from Japan (red circles) group well with the cloud of *P*. *issikii* specimens from the Russian Far East and South Korea (Wilks’ λ = 0.55, *p* = 0.21, N = 16).

Discriminant analysis based on the morphometry of four genital characters (length of left valva, left spine, right spine and phallus) indicated discrepancy in the analyzed dataset and proved the presence of two clusters (Wilks' λ = 0.27, F (4; 47) = 34.02, *p* < 0.0001, APCC–average percent of correct classification = 98%). Two out of four genital characters (i.e. length of right spine and phallus) played curial role in delineation of these clusters. One cluster was represented by all specimens of *P*. *issikii* from the presumable invaded regions (Bulgaria, Finland, Hungary, Italy, Poland, Western Russia and Siberia) and by four specimens of *P*. *issikii* from Japan (Fig 9). Important to mention that four Japanese individuals, which entered the first cluster, all carried the dominant haplotype H1 (red), which was widely present in Europe. The second cluster exclusively consisted of East Asian specimens, i.e. 14 specimens of *P*. *issikii* from the Russian Far East and from South Korea. Due to the lack of males of the putative new species, only two specimens, originating from Japan, were included into analysis. They well grouped with the second cluster of *P*. *issikii* from East Asia (Wilks’ λ = 0.55, *p* = 0.21, N = 16) (Fig 9).

**Fig 10:**
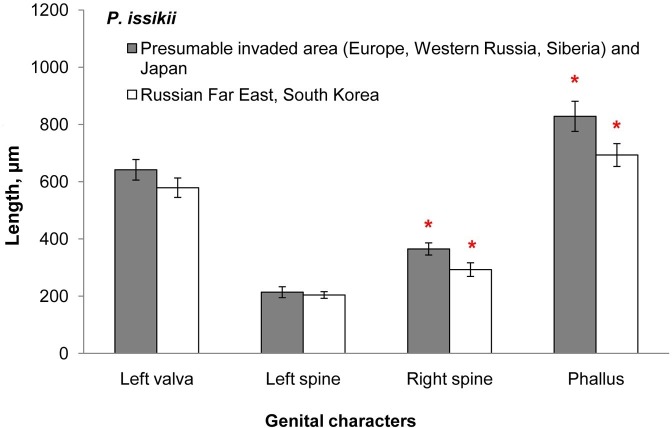
Mean values (± standard deviation) of male genital characters in *Phyllonorycter issikii* from various geographical populations. Data from the presumable invaded area (Europe, Western Russia and Siberia) and Japan were pooled together due to absence of significant difference between localities in the dataset (Wilks’ λ = 0.47, *p* = 0.28, N = 38); same was done for the data from the Russian Far East and South Korea (Wilks λ = 0.68, *p* = 0.44, N = 14). The bars with red asterisk are significant: the right spine (partial Wilks’ λ = 0.75, *p* = 0.0002, N = 52) and the phallus (partial Wilks’ λ = 0.87, *p* = 0.013, N = 52).

Right spine and phallus were about 17% shorter in *P*. *issikii* specimens originating from East Asia comparing with those from the presumable invaded area and Japan (right spine: partial Wilks’ λ = 0.75, *p* = 0.0002, N = 52; phallus: partial Wilks’ λ = 0.87, *p* = 0.013, N = 52; Fig 10). Length of left valva and length of left spine were not different between the two clusters of *P*. *issikii* (left valva: partial Wilks’ λ = 0.97, *p* = 0.28, N = 52; left spine: partial Wilks’ λ = 0.93, *p* = 0.06, N = 52; see also Fig 10).

## Discussion

Biological invasions occur when species become established in a new range outside the area of origin in which they proliferate and spread [58]. During the process of invasion there is usually a reduction of genetic diversity in the invaded ranges as a result of founder effects [59]. Indeed this loss of genetic diversity in the invaded region compared to the native areas has been shown in several invasive insects in general [60, 61] and Lepidoptera in particular [9, 10, 59].

Our study revealed an unexpected result in the populations of an invasive leaf mining micromoth: among 31 haplotypes of *Phyllonorycter issikii* discovered across the Palearctic, 23 haplotypes were present in the presumable invasive area (Europe, West Russia and Siberia), whereas only 10 haplotypes were detected in the presumable native range in East Asia (the Russian Far East, Japan, South Korea and China). Although the number of sequenced individuals was biased to the presumable invaded areas, the calculated means of haplotype diversities, which take into account number of the studied specimens, showed similar genetic diversity between the putative invasive and native ranges, supporting that a reduction of genetic diversity has not occurred at the expansion.

Only two haplotypes, the dominant haplotype H1 and sub-dominant H8 were shared between the presumable invaded range and the presumable native range. Interestingly the haplotype H1, most commonly found in the presumable invaded area (represented by 40% individuals in Europe, Western Russia and Siberia) was detected in 96% of insect individuals originating from Japan, suggesting possible contribution of Japan to *P*. *issikii* invasion westwards. Dominance of one haplotype in the invaded area was also found in another invasive gracillariid, *Cameraria ohridella* [9, 10].

The total number of haplotypes discovered for *P*. *issikii* in our study (31 haplotypes for 334 specimens analysed) is relatively high compared with that found for *C*. *ohridella* (28 haplotypes for 577 individuals analysed) [9, 10]. But what is more surprising is the high number of *P*. *issikii* haplotypes in the presumable invaded area (23 haplotypes) compared to the *Cameraria* study (3 haplotypes).

An explanation for the observed haplotype diversity of *P*. *issikii* in Europe, Western Russia and Siberia might be multiple introductions of the leaf miner from East Asia. The star-like patterns of haplotype clusters in *P*. *issikii* populations on the west of the Palearctic (see Fig 6) could be an evidence of such hybridization and radiation.

The majority of *P*. *issikii* haplotypes found on the west, might remain undiscovered in East Asia, as the centre of genetic diversity of *P*. *issikii* might present not in Japan and the Russian Far East, most sampled in our study, but in other parts of East Asia, such as Korea and China. So far we succeeded to collect only few specimens of *P*. *issikii* in South Korea and China because of extremely low population density. Moreover, before our survey *P*. *issikii* was not even known in China.

Another alternative explanation is that *P*. *issikii* might be native for some parts of Europe and western Russia but has been overlooked for decades because of undetectable population densities. This suggestion is however unlikely. In Europe and in Western Russia fauna of Gracillariidae, including rare moth species, has received quite a lot of attention over the last century. In earlier literature and keys for these regions there were no any note on *Phyllonorycter* species developing exclusively on *Tilia* [62, 63, 64]. The lime leaf miner shows low levels of parasitism rates in Europe and Western Russia [18], which reinforces the idea that *P*. *issikii* is not native to these regions. In addition, in Western Siberia, where *Tilia* range is very fragmented, *P*. *issikii* is presently found in remote forests of wild *Tilia sibirica* Bayer, 1862 and *Tilia cordata* Miller, 1768, which remained since the pre-glacial period [65, 66, 67, 68]. Despite Siberia remaining a poorly studied region, these lime grooves are protected and have been under long-term monitoring programs [68, 69, 70, 71, 72], however *P*. *issikii* had never been observed there until recently [32, 73].

The population of *P*. *issikii* from the Russian Far East shows five unique haplotypes present in 95% of individuals. In addition, morphometric analysis of male genitalia pointed at some difference between individuals from Russian Far East and *P*. *issikii* from other parts of its present range (Europe, Western Russia, Siberia, Japan and South Korea). The genetic and morphometric divergence of *P*. *issikii* found in the Russian Far East could be the result of incipient speciation due to geographic isolation. The Sikhote-Alin mountain range of 900 km long, expanding from Vladivostok along the coast to north-east of Primorsky krai where *P*. *issikii* occurs is known as an area of endemism for number of species [74, 75] and may act as a barrier causing allopatric divergence.

Analysis of genetic variation within and among populations of an invasive species across its modern range can also help clarify taxonomic identity of a pest and to delimit new species [36, 76, 77]. Molecular data (both mitochondrial and nuclear) suggests presence of an undescribed cryptic species of *Phyllonorycter* in East Asia. Indeed, the minimum pairwise genetic distance between *P*. *issikii* and the putative new species (3.66%) is similar to what we found in other groups of *Phyllonorycter* [78]. Specimens of the putative new species, (mines with larvae and pupae), were collected together with those of *P*. *issikii*, as they were undistinguishable. Despite both species occur in the same locations and shared same host plants with *P*. *issikii* (particularly, *T*. *maximowicziana*, *T*. *japonica*, *T*. *amurensis*, *T*. *mandshurica* and *T*. *taquetii*), we recorded no case of introgression. Surprisingly, the putative new species is rather abundant in the Russian Far East and Japan. We found it in more locations than *P*. *issikii*. Thus, it has been overlooked in East Asia for decades and only DNA barcoding screening of *Tilia*-feeding *Phyllonorycter* revealed its presence.

So far we found no difference between the putative new species and *P*. *issikii* in external morphology. Morphometric data of male genitalia has been shown to be a powerful tool to distinguish morphologically similar species of Gracillariidae [79]. Unfortunately the majority of studied material (i.e. 41 out of 43 specimens) was represented by larvae of female moths and only two adult males of the putative new *Phyllonorycter* species were reared. More males are needed to carry out robust morphometric analyses to confirm the presence and describe the putative new cryptic species occurring in sympatry with *P*. *issikii* in the Russian Far East and Japan.

### Future directions

A deeper population genetic analysis based on next generation sequencing and involving more populations of *P*. *issikii* from Eastern Asia (particularly, from Korea and Eastern China) would be required to detect the spots of high genetic diversity of the lime leaf miner within local populations and to understand the putative speciation processes happening in *Tilia*-feeding *Phyllonorycter* complex in East Asia. The study of past herbaria collections from Europe could be an important source of early data on the lime leaf miner presence on *Tilia* in the western Palearctic.

A special attention has to be paid to the geographical isolation of *P*. *issikii* population in the Russian Far East, as well as to the co-occurrence of the putative new *Phyllonorycter* species with *P*. *issikii* in East Asia. The candidate species does not seem to have expanded its distribution range yet. Thus a comparative study of the highly invasive *P*. *issikii* and its newly discovered non-invasive sister taxon might be a good model to identify the ecological and evolutionary factors and life history traits associated with invasion success.

## Supporting information

S1 Fig Distribution of normalized COI sequence divergence (K2P) for species (blue) against the genus (red) divergences at *Phyllonorycter issikii* and the putative *Phyllonorycter* sp. n. This pdf file contains the data included in this manuscript.
(PDF)

S1 Table Specimens used for molecular and morphological analyses. The Process ID code is a unique identifier linking the record in the BOLD database and the voucher specimen from which the sequence is obtained. The detailed collecting and specimen data are accessible in the BOLD dataset (http://dx.doi.org/DS-TILIAPHY). This pdf file contains the data included in this manuscript.
(PDF)

S2 Table Presence of *Phyllonorycter issikii* haplotypes in different countries in the Palearctic. This pdf file contains the data included in this manuscript.
(PDF)

S3 Table Distribution of COI sequence divergences (K2P) at *Phyllonorycter issikii* and the putative *Phyllonorycter* sp. n. This pdf file contains the data included in this manuscript.
(PDF)
