# Supplementary material for: From east to west across the Palearctic: Phylogeography of the invasive lime leaf miner Phyllonorycter issikii (Lepidoptera: Gracillariidae) and discovery of a putative new cryptic species in East Asia
Source: PLoS One. 2017 Feb 10;12(2):e0171104. doi: 10.1371/journal.pone.0171104 (PMC5302804; doi:10.1371/journal.pone.0171104)

Supplementary material

S1 Fig. Distribution of normalized COI sequence divergence (K2P) for species (blue) against the genus (red) divergences at *Phyllonorycter issikii* and the putative *Phyllonorycter* sp. n.

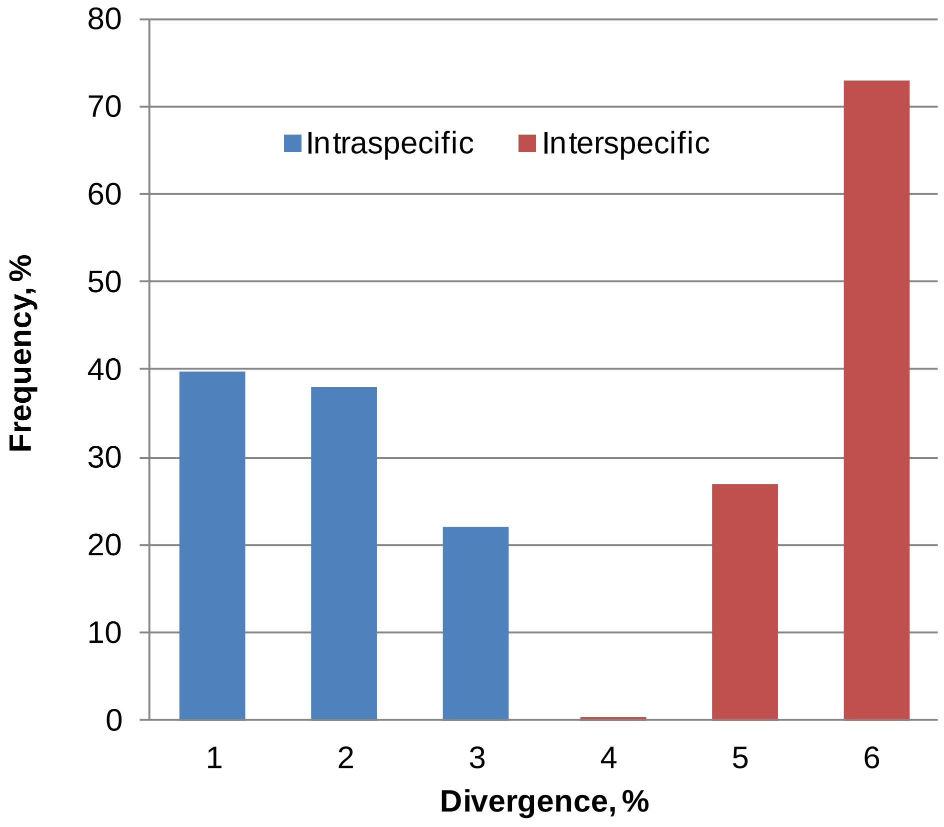

Supplement: S1 Fig — This pdf file contains the data included in this manuscript. (PDF) [file pone.0171104.s001.pdf]
