# Supplementary material for: From east to west across the Palearctic: Phylogeography of the invasive lime leaf miner Phyllonorycter issikii (Lepidoptera: Gracillariidae) and discovery of a putative new cryptic species in East Asia
Source: PLoS One. 2017 Feb 10;12(2):e0171104. doi: 10.1371/journal.pone.0171104 (PMC5302804; doi:10.1371/journal.pone.0171104)
Supplement: S1 Table — The Process ID code is a unique identifier linking the record in the BOLD database and the voucher specimen from which the sequence is obtained. The detailed collecting and specimen data are accessible in the BOLD dataset (http://dx.doi.org/DS-TILIAPHY). This pdf file contains the data included in this manuscript. (PDF) [file pone.0171104.s002.pdf]

Supplementary material

S1Table. Specimens used for molecular and morphological analyses[i]. The Process ID code is a unique identifier linking the record in the BOLD database and the voucher specimen from which the sequence is obtained. The detailed collecting and specimen data are accessible in the BOLD dataset (http://dx.doi.org/DS-TILIAPHY).

№	Species[1]	Insect stage[2], genitalia slide number	Process ID	Collectors	Host plant[3]	Country	Location[4]	Latitude	Longitude	Elevation	GenBank accession COI	GenBank accession H3	GenBank accession 28S
1.	*Phyllonorycter issikii*	A	GRSLO391-11	C. Wieser	—	Austria	Kaernten	46.5508	14.2017	570	JF848468	—	—
2.	*P. issikii*	A	GRSLO404-11	C. Wieser	—	Austria	Kaernten	46.5508	14.2017	570	JF848480	—	—
3.	*P. issikii*	A	GRSLO396-11	C. Wieser	—	Austria	Kaernten	46.5508	14.2017	570	JF848473	—	—
4.	*P. issikii*	A	GRSLO368-10	C. Wieser	—	Austria	Kaernten	46.5508	14.2017	570	JF848445	—	—
5.	*P. issikii*	A	GRSLO370-10	C. Wieser	—	Austria	Kaernten	46.5508	14.2017	570	JF848447	—	—
6.	*P. issikii*	A	GRSLO346-10	C. Wieser	—	Austria	Kaernten	46.5747	13.7639	550	JF848426	—	—
7.	*P. issikii*	A	GRSLO321-10	C. Wieser	—	Austria	Kaernten	46.5747	13.7639	550	JF848405	—	—
8.	*P. issikii*	A	GRSLO322-10	C. Wieser	—	Austria	Kaernten	46.5747	13.7639	550	JF848406	—	—
9.	*P. issikii*	A	GRSLO333-10	C. Wieser	—	Austria	Kaernten	46.5792	13.7433	570	JF848414	—	—
10.	*P. issikii*	A	GRSLO403-11	C. Wieser	—	Austria	Kaernten	46.6303	14.1828	530	JF848479	—	—
11.	*P. issikii*	A	GRSLO631-11	C. Wieser	—	Austria	Kaernten	46.6311	14.1811	530	JN299440	—	—
12.	*P. issikii*	A	GRSLO389-11	C. Wieser	—	Austria	Kaernten	46.7264	14.5344	510	JF848466	—	—
13.	*P. issikii*	A	GRSLO613-11	C. Wieser	—	Austria	Kaernten	46.7264	14.5344	510	JN299423	—	—
14.	*P. issikii*	A	GRSLO376-10	C. Wieser	—	Austria	Kaernten	46.7264	14.5344	510	JF848453	—	—
15.	*P. issikii*	A	GRSLO077-10	H. Deutsch	*Tilia platyphyllos*	Austria	Osttirol	46.8008	12.8839	725	JF848225	—	—
16.	*P. issikii*	A	GRSLO358-10	G. Stangelmaier	—	Austria	Kaernten	46.9100	13.9461	198	JF848436	—	—
17.	*P. issikii*	A	LEATF088-14	P. Huemer	—	Austria	Nordtirol	47.1570	10.9230	110	KY079349	—	—
18.	*P. issikii*	A	LEATE723-13	P. Huemer	—	Austria	Tirol	47.1760	10.6040	880	KY079351	—	—
19.	*P. issikii*	A	LEATH143-14	P. Huemer	—	Austria	Nordtirol	47.2200	10.8630	780	KY079345	—	—
20.	*P. issikii*	A	LEATH142-14	P. Huemer	—	Austria	Nordtirol	47.2200	10.8630	780	KY079346	—	—
21.	*P. issikii*	A	GRPAL1239-13	G. Deschka	*T. cordata*	Austria	—	48.0510	14.4146	330	KX818538	—	—
22.	*P. issikii*	A	GRPAL1238-13	G. Deschka	*T. platyphyllos*	Austria	—	48.0510	14.4146	330	KX818540	—	—

№	Species[1]	Insect stage[2], genitalia slide number	Process ID	Collectors	Host plant[3]	Country	Location[4]	Latitude	Longitude	Elevation	GenBank accession COI	GenBank accession H3	GenBank accession 28S
23.	*P. issikii*	A	GRPAL1234-13	G. Deschka	*T. platyphyllos*	Austria	—	48.0510	14.4146	330	KX818541	—	—
24.	*P. issikii*	A	GRPAL1236-13	G. Deschka	*T. platyphyllos*	Austria	—	48.0510	14.4146	330	KX818544	—	—
25.	*P. issikii*	A	GRPAL1237-13	G. Deschka	*T. platyphyllos*	Austria	—	48.0510	14.4146	330	KX818528	—	—
26.	*P. issikii*	A	GRPAL1240-13	G. Deschka	*T. platyphyllos*	Austria	—	48.0510	14.4146	330	KX818527	—	—
27.	*P. issikii*	A	GRPAL1232-13	G. Deschka	*T. platyphyllos*	Austria	—	48.0510	14.4146	330	KX818533	—	—
28.	*P. issikii*	A	GRPAL1231-13	G. Deschka	*T. platyphyllos*	Austria	—	48.0510	14.4146	330	KX818525	—	—
29.	*P. issikii*	A	GRPAL1230-13	G. Deschka	*T. cordata*	Austria	Kronstorf	48.1591	14.3955	300	KX818539	—	—
30.	*P. issikii*	A	GRPAL1233-13	G. Deschka	*T. cordata*	Austria	Kronstorf	48.1591	14.3955	300	KX818535	—	—
31.	*P. issikii*	A	ISSIK203-14	N. Kirichenko	*T. cordata*	Bulgaria	Sofia	42.6828	23.3369	562	KX818682	—	—
32.	*P. issikii*	A	ISSIK199-14	N. Kirichenko	*T. cordata*	Bulgaria	Sofia	42.6828	23.3369	562	KX818678	—	—
33.	*P. issikii*	A	ISSIK196-14	N. Kirichenko	*T. cordata*	Bulgaria	Sofia	42.6828	23.3369	562	KX818675	—	—
34.	*P. issikii*	A	LNOUD290-11	C. Lopez-Vaamonde	*Tilia sp.*	Bulgaria	—	42.6833	23.3167	400	KX044321	—	—
35.	*P. issikii*	A	LNOUD291-11	C. Lopez-Vaamonde	*Tilia sp.*	Bulgaria	—	42.6833	23.3167	400	KX047132	—	—
36.	*P. issikii*	A*, Bul-1♂	ISSIK201-14	N. Kirichenko	*T. cordata*	Bulgaria	Sofia	42.6828	23.3369	562	KX818680	—	—
37.	*P. issikii*	A*, Bul-2♂	ISSIK198-14	N. Kirichenko	*T. cordata*	Bulgaria	Sofia	42.6828	23.3369	562	KX818677	—	—
38.	*P. issikii*	A*, Bul-3♂	ISSIK197-14	N. Kirichenko	*T. cordata*	Bulgaria	Sofia	42.6828	23.3369	562	KX818676	—	—
39.	*P. issikii*	A*, Bul-27♂	ISSIK200-14	N. Kirichenko	*T. cordata*	Bulgaria	Sofia	42.6828	23.3369	562	KX818679	—	—
40.	*P. issikii*	A*, Bul-28♂	ISSIK202-14	N. Kirichenko	*T. cordata*	Bulgaria	Sofia	42.6828	23.3369	562	KX818681	—	—
41.	*P. issikii*	L	ISSIK264-14	N. Kirichenko	*T. cordata*	Bulgaria	Sofia	42.6830	23.337	562	KX818597	—	—
42.	*P. issikii*	L	ISSIK263-14	N. Kirichenko	*T. cordata*	Bulgaria	Sofia	42.6830	23.3370	562	KX818596	—	—
43.	*P. issikii*	L	ISSIK265-14	N. Kirichenko	*T. cordata*	Bulgaria	Sofia	42.6830	23.3370	562	KX818598	—	—
44.	*P. issikii*	L	ISSIK085-14	N. Kirichenko	*T. cordata*	Bulgaria	Sofia	42.6830	23.3370	562	KX818634	—	—
45.	*P. issikii*	L	ISSIK086-14	N. Kirichenko	*T. cordata*	Bulgaria	Sofia	42.6830	23.3370	562	KX818635	—	—
46.	*P. issikii*	P	ISSIK266-14	N. Kirichenko	*T. cordata*	Bulgaria	Sofia	42.6830	23.3370	562	KX818599	—	—
47.	*P. issikii*	P	ISSIK337-14	N. Kirichenko	*T. cordata*	Bulgaria	Sofia	42.6830	23.3370	562	KX818575	—	—
48.	*P. issikii*	P	ISSIK338-14	N. Kirichenko	*T. cordata*	Bulgaria	Sofia	42.6830	23.3370	562	KX818574	—	—
49.	*P. issikii*	P	ISSIK339-14	N. Kirichenko	*T. cordata*	Bulgaria	Sofia	42.6830	23.3370	562	KX818573	—	—

№	Species[1]	Insect stage[2], genitalia slide number	Process ID	Collectors	Host plant[3]	Country	Location[4]	Latitude	Longitude	Elevation	GenBank accession COI	GenBank accession H3	GenBank accession 28S
50.	*P. issikii*	P	ISSIK087-14	N. Kirichenko	*T. cordata*	Bulgaria	Sofia	42.6830	23.3370	562	KX818636	—	—
51.	*P. issikii*	P	ISSIK336-14	N. Kirichenko	*T. cordata*	Bulgaria	Sofia	42.6830	23.3370	562	KX818450	—	—
52.	*P. issikii*	P	ISSIK335-14	N. Kirichenko	*T. cordata*	Bulgaria	Sofia	42.6830	23.3370	562	KX818451	—	—
53.	*P. issikii*	L	MICRU019-15	T. Liu	*T. mongolica*	China	Tianjin	40.0920	117.4370	207	KX818524	KX818870	KX818755
54.	*P. issikii*	A	GRSLO529-11	Jan Liska	*T. platyphyllos*	Czech Republic	Prague	50.1080	14.424	186	JN280352	—	—
55.	*P. issikii*	A*, Fin-10♂	ISSIK215-14	M. Mutanen	*T. cordata*	Finland	Salo	60.3069	23.3086	70	KX818472	—	—
56.	*P. issikii*	A*, Fin-12♂	ISSIK216-14	M. Mutanen	*T. cordata*	Finland	Salo	60.3069	23.3086	70	KX818473	—	—
57.	*P. issikii*	A*, Fin-13♂	ISSIK217-14	M. Mutanen	*T. cordata*	Finland	Turku	60.4492	22.2542	10	KX818474	—	—
58.	*P. issikii*	A*, Fin-38♂	ISSIK218-14	M. Mutanen	*T. cordata*	Finland	Salo	60.3069	23.3086	70	KX818475	—	—
59.	*P. issikii*	A	ISSIK088-14	M. Mutanen, J. Itamies	*T. cordata*	Finland	Salo	60.3070	23.3090	70	KX818637	—	—
60.	*P. issikii*	A	ISSIK089-14	M. Mutanen, J. Itamies	*T. cordata*	Finland	Salo	60.3070	23.3090	70	KX818638	—	—
61.	*P. issikii*	A	ISSIK090-14	M. Mutanen, J. Itamies	*T. cordata*	Finland	Salo	60.3070	23.3090	70	KX818639	—	—
62.	*P. issikii*	A	ISSIK091-14	M. Mutanen, J. Itamies	*T. cordata*	Finland	Salo	60.3070	23.3090	70	KX818640	—	—
63.	*P. issikii*	A	ISSIK219-14	M. Mutanen	*T. cordata*	Finland	Salo	60.3069	23.3086	70	KX818476	—	—
64.	*P. issikii*	A	ISSIK220-14	M. Mutanen	*T. cordata*	Finland	Salo	60.3069	23.3086	70	KX818477	—	—
65.	*P. issikii*	A	ISSIK327-14	M. Mutanen	*T. cordata*	Finland	Salo	60.3070	23.3090	70	KX818459	—	—
66.	*P. issikii*	A	ISSIK328-14	M. Mutanen	*T. cordata*	Finland	Salo	60.3070	23.3090	70	KX818458	—	—
67.	*P. issikii*	A	ISSIK329-14	M. Mutanen	*T. cordata*	Finland	Salo	60.3070	23.3090	70	KX818457	—	—
68.	*P. issikii*	A	LEFIB340-10	M. Mutanen, T. Mutanen	—	Finland	Regio Aboensis	60.4100	22.0980	12	HM871240	—	—
69.	*P. issikii*	A	LEFIB341-10	M. Mutanen, T. Mutanen	—	Finland	Regio Aboensis	60.4100	22.0980	12	HM871241	—	—
70.	*P. issikii*	A	LEFIB362-10	M. Mutanen, T. Mutanen	—	Finland	Regio Aboensis	60.4100	22.0980	12	HM871261	—	—
71.	*P. issikii*	A	LEFIJ2189-14	T. Mutanen	—	Finland	Regio aboensis	60.3495	23.1135	12	KT782643	—	—
72.	*P. issikii*	A	FGMLC241-13	A. H. Segerer	—	Germany	Oberbayern	48.1674	11.4935	520	KY079354	—	—
73.	*P. issikii*	A	FGMLA321-12	T. Gruenewald	—	Germany	Niederbayern	48.5289	12.1165	400	KY079353	—	—
74.	*P. issikii*	A	ODOPE146-11	P. Lichtmannecker	—	Germany	Niederbayern	48.5871	13.5330	375	KX040764	—	—

№	Species[1]	Insect stage[2], genitalia slide number	Process ID	Collectors	Host plant[3]	Country	Location[4]	Latitude	Longitude	Elevation	GenBank accession COI	GenBank accession H3	GenBank accession 28S
75.	*P. issikii*	A	FBLMZ040-12	R. Heindel	—	Germany	Schwaben	48.8038	10.6453	470	KY079343	—	—
76.	*P. issikii*	A	ISSIK233-14	L. Szocs	*T. cordata*	Hungary	Zalakomar	46.5350	17.1731	118	KX818490	—	—
77.	*P. issikii*	A*, Hun 29♂	ISSIK231-14	L. Szocs	*T. cordata*	Hungary	Zalakomar	46.5350	17.1731	118	KX818488	—	—
78.	*P. issikii*	A*, Hun 30♂	ISSIK232-14	L. Szocs	*T. cordata*	Hungary	Zalakomar	46.5350	17.1731	118	KX818489	—	—
79.	*P. issikii*	A*	ISSIK230-14	L. Szocs	*T. cordata*	Hungary	Zalakomar	46.5350	17.1731	118	KX818487	—	—
80.	*P. issikii*	A*	ISSIK343-14	L. Szocs	*T. cordata*	Hungary	Bajansenye	46.7981	16.3853	222	KX818569	—	—
81.	*P. issikii*	A*	ISSIK344-14	L. Szocs	*T. cordata*	Hungary	Nemeshany	47.0677	17.3642	160	KX818568	—	—
82.	*P. issikii*	A*	ISSIK333-14	L. Szocs	*T. cordata*	Hungary	Kam	47.1081	16.8918	169	KX818453	—	—
83.	*P. issikii*	A*	ISSIK334-14	L. Szocs	*T. cordata*	Hungary	Kam	47.1081	16.8918	169	KX818452	—	—
84.	*P. issikii*	A*	ISSIK332-14	L. Szocs	*T. cordata*	Hungary	Kam	47.1081	16.8918	169	KX818454	—	—
85.	*P. issikii*	A	ISSIK347-14	L. Szocs	*T. cordata*	Hungary	Marko	47.1193	17.8152	374	KX818565	—	—
86.	*P. issikii*	A	ISSIK331-14	L. Szocs	*T. cordata*	Hungary	Csehbanya	47.1734	17.6748	460	KX818455	—	—
87.	*P. issikii*	A	ISSIK330-14	L. Szocs	*T. cordata*	Hungary	Csehbanya	47.1734	17.6748	460	KX818456	—	—
88.	*P. issikii*	A	ISSIK227-14	L. Szocs	*T. platyphyllos*	Hungary	Farkasgyepu	47.2047	17.6283	386	KX818484	—	—
89.	*P. issikii*	A	ISSIK228-14	L. Szocs	*T. platyphyllos*	Hungary	Farkasgyepu	47.2047	17.6283	386	KX818485	—	—
90.	*P. issikii*	A	ISSIK229-14	L. Szocs	*T. platyphyllos*	Hungary	Farkasgyepu	47.2047	17.6283	386	KX818486	—	—
91.	*P. issikii*	A	ISSIK226-14	L. Szocs	*T. platyphyllos*	Hungary	Farkasgyepu	47.2047	17.6283	386	KX818483	—	—
92.	*P. issikii*	A	ISSIK342-14	L. Szocs	*T. cordata*	Hungary	Sarvar	47.2490	16.9295	154	KX818570	—	—
93.	*P. issikii*	A	ISSIK346-14	L. Szocs	*T. cordata*	Hungary	Budapest	47.5016	19.0341	167	KX818566	—	—
94.	*P. issikii*	A	ISSIK341-14	L. Szocs	*T. cordata*	Hungary	Szilvasvarad	48.0936	20.3820	354	KX818571	—	—
95.	*P. issikii*	A	ISSIK340-14	L. Szocs	*T. cordata*	Hungary	Szilvasvarad	48.0936	20.3820	354	KX818572	—	—
96.	*P. issikii*	A	ISSIK345-14	L. Szocs	*T. cordata*	Hungary	Vagashuta	48.4224	21.5449	295	KX818567	—	—
97.	*P. issikii*	A	PHLAD034-11	P. Huemer	—	Italy	Etschtal	46.4283	11.3000	643	JN280415	—	—
98.	*P. issikii*	A	LEATH372-14	P. Huemer	—	Italy	Suedtirol	46.7490	10.6650	840	KY079347	—	—
99.	*P. issikii*	A*, IT 3976♂	—	P. Triberti	—	Italy	Bressanone	46.4300	11.3900	560	—	—	—
100.	*P. issikii*	A*, IT 3978♂	—	P. Triberti	—	Italy	Bressanone	46.4300	11.3900	560	—	—	—
101.	*P. issikii*	A*, IT	—	P. Triberti	—	Italy	Bressanone	46.4300	11.3900	560	—	—	—

№	Species[1]	Insect stage[2], genitalia slide number	Process ID	Collectors	Host plant[3]	Country	Location[4]	Latitude	Longitude	Elevation	GenBank accession COI	GenBank accession H3	GenBank accession 28S
		3979♂											
102.	*P. issikii*	A*, IT 3980♂	—	P. Triberti	—	Italy	Bressanone	46.4300	11.3900	560	—	—	—
103.	*P. issikii*	A*, IT 3987♂	—	P. Triberti	—	Italy	Bressanone	46.4300	11.3900	560	—	—	—
104.	*P. issikii*	A*, IT 3989♂	—	P. Triberti	—	Italy	Bressanone	46.4300	11.3900	560	—	—	—
105.	*P. issikii*	A*, IT 31♂	—	P. Triberti	—	Italy	Bressanone	46.4300	11.3900	560	—	—	—
106.	*P. issikii*	A*, IT 32♂	—	P. Triberti	—	Italy	Bressanone	46.4300	11.3900	560	—	—	—
107.	*P. issikii*	A*, IT 33♂	—	P. Triberti	—	Italy	Bressanone	46.4300	11.3900	560	—	—	—
108.	*P. issikii*	A*, SaPPo 01♂	ISSIK301-14	T. Kanbe	*T. maximowicziana*	Japan	Sapporo	43.0340	141.3150	121	KX818408	—	—
109.	*P. issikii*	A*, SaPPo 06♂	ISSIK304-14	T. Kanbe	*T. maximowicziana*	Japan	Sapporo	43.0340	141.3150	121	KX818405	KX818850	KX818735
110.	*P. issikii*	A*, SaPPo 12♂	ISSIK312-14	T. Kanbe	*T. maximowicziana*	Japan	Sapporo	43.0340	141.3150	121	KX818402	—	—
111.	*P. issikii*	A*, SaPPo 25♂	ISSIK322-14	T. Kanbe	*T. maximowicziana*	Japan	Sapporo	43.0340	141.3150	121	KX818462	KX818865	KX818750
112.	*P. issikii*	A*, SaPPo 26♂	ISSIK324-14	T. Kanbe	*T. maximowicziana*	Japan	Sapporo	43.0340	141.3150	121	KX818460	KX818864	KX818749
113.	*P. issikii*	A	ISSIK310-14	T. Kanbe	*T. maximowicziana*	Japan	Sapporo	43.0340	141.3150	121	KX818404	KX818849	KX818734
114.	*P. issikii*	A	ISSIK302-14	T. Kanbe	*T. maximowicziana*	Japan	Sapporo	43.0340	141.3150	121	KX818407	KX818852	KX818737
115.	*P. issikii*	A	ISSIK297-14	T. Kanbe	*T. maximowicziana*	Japan	Sapporo	43.0340	141.3150	121	KX818411		
116.	*P. issikii*	A	ISSIK298-14	T. Kanbe	*T. maximowicziana*	Japan	Sapporo	43.0340	141.3150	121	KX818410	KX818854	KX818739
117.	*P. issikii*	A	ISSIK300-14	T. Kanbe	*T. maximowicziana*	Japan	Sapporo	43.0340	141.3150	121	KX818409	KX818853	KX818738
118.	*P. issikii*	A	ISSIK311-14	T. Kanbe	*T. maximowicziana*	Japan	Sapporo	43.0340	141.3150	121	KX818403		
119.	*P. issikii*	A	ISSIK317-14	T. Kanbe	*T. maximowicziana*	Japan	Sapporo	43.0340	141.3150	121	KX818397	KX818845	KX818730
120.	*P. issikii*	A	ISSIK316-14	T. Kanbe	*T. maximowicziana*	Japan	Sapporo	43.0340	141.3150	121	KX818398	KX818846	KX818731
121.	*P. issikii*	A	ISSIK315-14	T. Kanbe	*T. maximowicziana*	Japan	Sapporo	43.0340	141.3150	121	KX818399	KX818847	KX818732
122.	*P. issikii*	A	ISSIK313-14	T. Kanbe	*T. maximowicziana*	Japan	Sapporo	43.0340	141.3150	121	KX818401	KX818848	KX818733
123.	*P. issikii*	A	ISSIK314-14	T. Kanbe	*T. maximowicziana*	Japan	Sapporo	43.0340	141.3150	121	KX818400	—	—
124.	*P. issikii*	A	ISSIK323-14	T. Kanbe	*T. maximowicziana*	Japan	Sapporo	43.0340	141.3150	121	KX818461	—	—
125.	*P. issikii*	A	GRAAM048-13	G. Deschka	*T. maximowicziana*	Japan	Sapporo	43.1136	140.4530	30	KX818530	—	—
126.	*P. issikii*	A	GRAAM050-13	G. Deschka	*T. japonica*	Japan	Sapporo	43.0336	141.3320	58	KX818536	—	—

№	Species[1]	Insect stage[2], genitalia slide number	Process ID	Collectors	Host plant[3]	Country	Location[4]	Latitude	Longitude	Elevation	GenBank accession COI	GenBank accession H3	GenBank accession 28S
127.	*P. issikii*	L	ISSIK375-14	T. Kanbe	*T. maximowicziana*	Japan	Sapporo	43.0338	141.3150	121	KX818549	—	—
128.	*P. issikii*	L	ISSIK376-14	T. Kanbe	*T. maximowicziana*	Japan	Sapporo	43.0338	141.3150	121	KX818548	—	—
129.	*P. issikii*	L	ISSIK377-14	T. Kanbe	*T. maximowicziana*	Japan	Sapporo	43.0338	141.3150	121	KX818547	—	—
130.	*P. issikii*	L	ISSIK303-14	T. Kanbe	*T. maximowicziana*	Japan	Sapporo	43.0340	141.3150	121	KX818406	KX818851	KX818736
131.	*P. issikii*	A	GRACI473-09	P. Ivinskis	*T. platyphyllos*	Lithuania	Vilnius	54.6290	25.2820	165	HM392461	—	—
132.	*P. issikii*	A	GRPAL905-12	Buda	*T. platyphyllos*	Lithuania	Vilnius	54.6800	25.2800	150	KF367692	—	—
133.	*P. issikii*	A	GRPAL907-12	P. Ivinskis	*T. platyphyllos*	Lithuania	Vilnius	54.6800	25.2800	150	KF367709	—	—
134.	*P. issikii*	A	MPEA294-08	J. & W. De Prins	—	Lithuania	Vilnius	54.6900	25.2800	150	KY079352	—	—
135.	*P. issikii*	A	NLLEA1337-14	B. van As & Jan	*T. cordata*	Netherlands	Utrecht	51.9350	5.1100	13	KY079350	—	—
136.	*P. issikii*	A	NLGRA001-13	A. Schreurs	*Tilia sp.*	Netherlands	Limburg	50.7690	5.6250	138	KY079344	—	—
137.	*P. issikii*	A	GRACI385-09	A. Lastuvka	*Tilia sp.*	Poland	—	52.7725	23.8494	99	KX071164	—	—
138.	*P. issikii*	A	GRACI384-09	A. Lastuvka	*Tilia sp.*	Poland	—	52.7725	23.8494	99	KX070945		
139.	*P. issikii*	A*, POL34♂	—	W. May	—	Poland	Bialowieza	52.4570	23.5240	101	—	—	—
140.	*P. issikii*	L	ISSIK288-14	C. Lopez-Vaamonde	*Tilia sp.*	Poland	SEkocin Stary	52.1020	20.8820	121	KX818499	—	—
141.	*P. issikii*	L	ISSIK287-14	C. Lopez-Vaamonde	*Tilia sp.*	Poland	SEkocin Stary	52.1020	20.8820	121	KX818498	—	—
142.	*P. issikii*	L	ISSIK058-14	N. Kirichenko	*T. amurensis*	Russia	Barnaul	53.2920	83.7690	201	KX818610	—	—
143.	*P. issikii*	L	ISSIK057-14	N. Kirichenko	*T. amurensis*	Russia	Barnaul	53.2920	83.7690	201	KX818609	—	—
144.	*P. issikii*	L	ISSIK059-14	N. Kirichenko	*T. amurensis*	Russia	Barnaul	53.2920	83.7690	201	KX818611	—	—
145.	*P. issikii*	L	ISSIK063-14	N. Kirichenko	*T. cordata*	Russia	Barnaul	53.2920	83.7690	201	KX818615	—	—
146.	*P. issikii*	L	ISSIK062-14	N. Kirichenko	*T. cordata*	Russia	Barnaul	53.2920	83.7690	201	KX818614	—	—
147.	*P. issikii*	L	ISSIK061-14	N. Kirichenko	*T. cordata*	Russia	Barnaul	53.2920	83.7690	201	KX818613	—	—
148.	*P. issikii*	L	ISSIK060-14	N. Kirichenko	*T. cordata*	Russia	Barnaul	53.2920	83.7690	201	KX818612	—	—
149.	*P. issikii*	L	ISSIK239-14	N. Kirichenko	*T. cordata*	Russia	Barnaul	53.2920	83.7690	201	KX818518	—	—
150.	*P. issikii*	L	ISSIK054-14	N. Kirichenko	*T. sibirica*	Russia	Kuzedeevo	53.3360	87.2060	266	KX818606	—	—
151.	*P. issikii*	L	ISSIK055-14	N. Kirichenko	*T. sibirica*	Russia	Kuzedeevo	53.3360	87.2060	266	KX818607	—	—
152.	*P. issikii*	L	ISSIK056-14	N. Kirichenko	*T. sibirica*	Russia	Kuzedeevo	53.3360	87.2060	266	KX818608	—	—
153.	*P. issikii*	L	ISSIK361-14	N. Kirichenko	*T. sibirica*	Russia	Kuzedeevo	53.3360	87.2060	266	KX818551	—	—

№	Species[1]	Insect stage[2], genitalia slide number	Process ID	Collectors	Host plant[3]	Country	Location[4]	Latitude	Longitude	Elevation	GenBank accession COI	GenBank accession H3	GenBank accession 28S
154.	*P. issikii*	L	ISSIK362-14	N. Kirichenko	*T. sibirica*	Russia	Kuzedeevo	53.3360	87.2060	266	KX818550	—	—
155.	*P. issikii*	L	ISSIK237-14	N. Kirichenko	*T. sibirica*	Russia	Kuzedeevo	53.3360	87.2060	266	KX818516	—	—
156.	*P. issikii*	L	ISSIK238-14	N. Kirichenko	*T. sibirica*	Russia	Kuzedeevo	53.3360	87.2060	266	KX818517	—	—
157.	*P. issikii*	A*, WRus 14♂	ISSIK212-14	N. Kirichenko	*T. dasystyla*	Russia	Moscow	55.7419	37.6203	129	KX818469	—	—
158.	*P. issikii*	A*, WRus 15♂	ISSIK213-14	N. Kirichenko	*T. dasystyla*	Russia	Moscow	55.7419	37.6203	129	KX818470	—	—
159.	*P. issikii*	A*, WRus 16♂	ISSIK207-14	N. Kirichenko	*T. cordata*	Russia	Moscow	55.7269	37.6117	137	KX818686	—	—
160.	*P. issikii*	A*, WRus 17♂	ISSIK211-14	N. Kirichenko	*T. dasystyla*	Russia	Moscow	55.7419	37.6203	129	KX818468	—	—
161.	*P. issikii*	A*, WRus 18♂	ISSIK208-14	N. Kirichenko	*T. cordata*	Russia	Moscow	55.7269	37.6117	137	KX818465	—	—
162.	*P. issikii*	A*, WRus 40♂	ISSIK209-14	N. Kirichenko	*T. dasystyla*	Russia	Moscow	55.7419	37.6203	129	KX818466	—	—
163.	*P. issikii*	A*, WRus 41♂	ISSIK206-14	N. Kirichenko	*T. cordata*	Russia	Moscow	55.7269	37.6117	137	KX818685	—	—
164.	*P. issikii*	A	ISSIK204-14	N. Kirichenko	*T. cordata*	Russia	Moscow	55.7269	37.6117	137	KX818683	—	—
165.	*P. issikii*	A	ISSIK205-14	N. Kirichenko	*T. cordata*	Russia	Moscow	55.7269	37.6117	137	KX818684	—	—
166.	*P. issikii*	A	ISSIK210-14	N. Kirichenko	*T. dasystyla*	Russia	Moscow	55.7419	37.6203	129	KX818467	—	—
167.	*P. issikii*	A	ISSIK214-14	N. Kirichenko	*T. dasystyla*	Russia	Moscow	55.7419	37.6203	129	KX818471	—	—
168.	*P. issikii*	L	ISSIK008-12	N. Kirichenko	*T. cordata*	Russia	Moscow	54.8390	37.6040	162	KX818421	—	—
169.	*P. issikii*	L	ISSIK007-12	N. Kirichenko	*T. cordata*	Russia	Moscow	54.8390	37.6040	162	KX818420	KX818858	KX818743
170.	*P. issikii*	L	ISSIK006-12	N. Kirichenko	*T. cordata*	Russia	Moscow	54.8390	37.6040	162	KX818419	—	—
171.	*P. issikii*	L	ISSIK005-12	N. Kirichenko	*T. cordata*	Russia	Moscow	54.8390	37.6040	162	KX818418	KX818857	KX818742
172.	*P. issikii*	L	ISSIK001-12	N. Kirichenko	*T. cordata*	Russia	Moscow	54.8390	37.6040	162	KX818414	—	—
173.	*P. issikii*	L	ISSIK004-12	N. Kirichenko	*T. cordata*	Russia	Moscow	54.8390	37.6040	162	KX818417	KX818856	KX818741
174.	*P. issikii*	L	ISSIK003-12	N. Kirichenko	*T. cordata*	Russia	Moscow	54.8390	37.6040	162	KX818416	—	—
175.	*P. issikii*	L	ISSIK002-12	N. Kirichenko	*T. cordata*	Russia	Moscow	54.8390	37.6040	162	KX818415	KX818855	KX818740
176.	*P. issikii*	L	ISSIK255-14	N. Kirichenko	*T. dasystyla*	Russia	Moscow	55.7420	37.6200	129	KX818588	—	—
177.	*P. issikii*	L	ISSIK254-14	N. Kirichenko	*T. dasystyla*	Russia	Moscow	55.7420	37.6200	129	KX818587	—	—
178.	*P. issikii*	L	ISSIK257-14	N. Kirichenko	*T. dasystyla*	Russia	Moscow	55.7420	37.6200	129	KX818590	—	—
179.	*P. issikii*	L	ISSIK258-14	N. Kirichenko	*T. dasystyla*	Russia	Moscow	55.7420	37.6200	129	KX818591	—	—

№	Species[1]	Insect stage[2], genitalia slide number	Process ID	Collectors	Host plant[3]	Country	Location[4]	Latitude	Longitude	Elevation	GenBank accession COI	GenBank accession H3	GenBank accession 28S
180.	*P. issikii*	L	ISSIK256-14	N. Kirichenko	*T. dasystyla*	Russia	Moscow	55.7420	37.6200	129	KX818589	—	—
181.	*P. issikii*	L	ISSIK259-14	J. Timofeeva	*T. cordata*	Russia	Saint Petersburg	59.9910	30.3420	23	KX818592	—	—
182.	*P. issikii*	L	ISSIK260-14	J. Timofeeva	*T. cordata*	Russia	Saint Petersburg	59.9910	30.3420	23	KX818593	—	—
183.	*P. issikii*	L	ISSIK262-14	J. Timofeeva	*T. cordata*	Russia	Saint Petersburg	59.9910	30.3420	23	KX818595	—	—
184.	*P. issikii*	L	ISSIK261-14	J. Timofeeva	*T. cordata*	Russia	Saint Petersburg	59.9910	30.3420	23	KX818594	—	—
185.	*P. issikii*	L	ISSIK084-14	J. Timofeeva	*T. cordata*	Russia	Saint Petersburg	59.9910	30.3420	23	KX818633	—	—
186.	*P. issikii*	L	ISSIK083-14	J. Timofeeva	*T. cordata*	Russia	Saint Petersburg	59.9910	30.3420	23	KX818632	—	—
187.	*P. issikii*	L	ISSIK296-14	J. Timofeeva	*T. cordata*	Russia	Saint Petersburg	59.9910	30.3420	23	KX818446	—	—
188.	*P. issikii*	L	ISSIK292-14	J. Timofeeva	*T. cordata*	Russia	Saint Petersburg	59.9910	30.3420	23	KX818503	—	—
189.	*P. issikii*	L	ISSIK291-14	J. Timofeeva	*T. cordata*	Russia	Saint Petersburg	59.9910	30.3420	23	KX818502	—	—
190.	*P. issikii*	L	ISSIK290-14	J. Timofeeva	*T. cordata*	Russia	Saint Petersburg	59.9910	30.3420	23	KX818501	—	—
191.	*P. issikii*	L	ISSIK293-14	J. Timofeeva	*T. cordata*	Russia	Saint Petersburg	59.9910	30.3420	23	KX818504	—	—
192.	*P. issikii*	L	ISSIK294-14	J. Timofeeva	*T. cordata*	Russia	Saint Petersburg	59.9910	30.3420	23	KX818505	—	—
193.	*P. issikii*	L	ISSIK295-14	J. Timofeeva	*T. cordata*	Russia	Saint Petersburg	59.9910	30.3420	23	KX818506	—	—
194.	*P. issikii*	L	ISSIK289-14	J. Timofeeva	*T. cordata*	Russia	Saint Petersburg	59.9910	30.3420	23	KX818500	—	—
195.	*P. issikii*	A*, WRus 37♂	ISSIK189-14	N. Kirichenko	*T. cordata*	Russia	Serpukhov	54.9619	37.4561	171	KX818670	—	—
196.	*P. issikii*	A*, WRus 36♂	ISSIK192-14	N. Kirichenko	*T. cordata*	Russia	Serpukhov	54.9619	37.4561	171	KX818673	—	—
197.	*P. issikii*	A*, WRus 35♂	ISSIK191-14	N. Kirichenko	*T. cordata*	Russia	Serpukhov	54.9619	37.4561	171	KX818672	—	—
198.	*P. issikii*	A*, WRus 42♂	ISSIK190-14	N. Kirichenko	*T. cordata*	Russia	Serpukhov	54.9619	37.4561	171	KX818671	—	—
199.	*P. issikii*	A	ISSIK195-14	N. Kirichenko	*T. cordata*	Russia	Serpukhov	54.9619	37.4561	171	KX818674	—	—
200.	*P. issikii*	L	ISSIK253-14	A. Bruykhanov	*T. cordata*	Russia	Serpukhov	54.9620	37.4560	171	KX818586	—	—
201.	*P. issikii*	L	ISSIK252-14	A. Bruykhanov	*T. cordata*	Russia	Serpukhov	54.9620	37.4560	171	KX818585	—	—
202.	*P. issikii*	L	ISSIK250-14	A. Bruykhanov	*T. cordata*	Russia	Serpukhov	54.9620	37.4560	171	KX818583	—	—
203.	*P. issikii*	L	ISSIK249-14	A. Bruykhanov	*T. cordata*	Russia	Serpukhov	54.9620	37.4560	171	KX818582	—	—
204.	*P. issikii*	L	ISSIK251-14	A. Bruykhanov	*T. cordata*	Russia	Serpukhov	54.9620	37.4560	171	KX818584	—	—
205.	*P. issikii*	L	ISSIK082-14	A. Bruykhanov	*T. cordata*	Russia	Serpukhov	54.9620	37.4560	171	KX818631	—	—
206.	*P. issikii*	A*, Sib-6♂	ISSIK223-14	N. Kirichenko	*T. cordata*	Russia	Novosibirsk	54.8200	83.1039	155	KX818480	—	—

№	Species[1]	Insect stage[2], genitalia slide number	Process ID	Collectors	Host plant[3]	Country	Location[4]	Latitude	Longitude	Elevation	GenBank accession COI	GenBank accession H3	GenBank accession 28S
207.	*P. issikii*	A*, Sib-7♂	ISSIK224-14	N. Kirichenko	*T. cordata*	Russia	Novosibirsk	54.8200	83.1039	155	KX818481	—	—
208.	*P. issikii*	A*, Sib-8♂	ISSIK222-14	N. Kirichenko	*T. cordata*	Russia	Novosibirsk	54.8200	83.1039	155	KX818479	—	—
209.	*P. issikii*	A	GRPAL1095-13	N. Kirichenko	*T. sibirica*	Russia	Novosibirsk	55.8358	83.1008	158	KX818537	—	—
210.	*P. issikii*	A	GRPAL1097-13	N. Kirichenko	*T. sibirica*	Russia	Novosibirsk	55.8358	83.1008	158	KX818542	—	—
211.	*P. issikii*	A	ISSIK188-14	N. Kirichenko	*T. cordata*	Russia	Novosibirsk	54.8200	83.1039	155	KX818669	—	—
212.	*P. issikii*	A	GRPAL1093-13	N. Kirichenko	*T. cordata*	Russia	Novosibirsk	55.8358	83.1008	158	KX818543	—	—
213.	*P. issikii*	A	GRACI471-09	N. Kirichenko	*T. sibirica*	Russia	Novosibirsk	54.0190	83.1410	220	HM392459	—	—
214.	*P. issikii*	A	GRPAL1094-13	N. Kirichenko	*T. cordata*	Russia	Novosibirsk	55.8358	83.1008	158	KX818529	—	—
215.	*P. issikii*	A	GRPAL1091-13	N. Kirichenko	*T. cordata*	Russia	Novosibirsk	55.8358	83.1008	158	KX818532	—	—
216.	*P. issikii*	A	GRACI468-09	N.Kirichenko	*T. cordata*	Russia	Novosibirsk	54.0190	83.1410	225	HM392456	—	—
217.	*P. issikii*	A	GRPAL1092-13	N. Kirichenko	*T. cordata*	Russia	Novosibirsk	55.8358	83.1008	158	KX818534	—	—
218.	*P. issikii*	A	GRPAL1096-13	N. Kirichenko	*T. sibirica*	Russia	Novosibirsk	55.8358	83.1008	158	KX818526	—	—
219.	*P. issikii*	A	ISSIK225-14	N. Kirichenko	*T. cordata*	Russia	Novosibirsk	54.8200	83.1039	155	KX818482	—	—
220.	*P. issikii*	A	ISSIK221-14	N. Kirichenko	*T. cordata*	Russia	Novosibirsk	54.8200	83.1039	155	KX818478	—	—
221.	*P. issikii*	L	ISSIK009-12	N. Kirichenko	*T. cordata*	Russia	Novosibirsk	54.8200	83.1040	155	KX818422	KX818859	KX818744
222.	*P. issikii*	L	ISSIK010-12	N. Kirichenko	*T. cordata*	Russia	Novosibirsk	54.8200	83.1040	155	KX818423	—	—
223.	*P. issikii*	L	ISSIK011-12	N. Kirichenko	*T. cordata*	Russia	Novosibirsk	54.8200	83.1040	155	KX818424	—	—
224.	*P. issikii*	L	ISSIK014-12	N. Kirichenko	*T. cordata*	Russia	Novosibirsk	54.8200	83.1040	155	KX818427	—	—
225.	*P. issikii*	L	ISSIK013-12	N. Kirichenko	*T. cordata*	Russia	Novosibirsk	54.8200	83.1040	155	KX818426	KX818860	KX818745
226.	*P. issikii*	L	ISSIK012-12	N. Kirichenko	*T. cordata*	Russia	Novosibirsk	54.8200	83.1040	155	KX818425	—	—
227.	*P. issikii*	L	ISSIK016-12	N. Kirichenko	*T. cordata*	Russia	Novosibirsk	54.8200	83.1040	155	KX818429	—	—
228.	*P. issikii*	L	ISSIK028-12	N. Kirichenko	*T. sibirica*	Russia	Novosibirsk	54.8200	83.1040	155	KX818441	—	—
229.	*P. issikii*	L	ISSIK027-12	N. Kirichenko	*T. sibirica*	Russia	Novosibirsk	54.8200	83.1040	155	KX818440	—	—
230.	*P. issikii*	L	ISSIK026-12	N. Kirichenko	*T. sibirica*	Russia	Novosibirsk	54.8200	83.1040	155	KX818439	—	—
231.	*P. issikii*	L	ISSIK025-12	N. Kirichenko	*T. sibirica*	Russia	Novosibirsk	54.8200	83.1040	155	KX818438	—	—
232.	*P. issikii*	L	ISSIK024-12	N. Kirichenko	*T. sibirica*	Russia	Novosibirsk	54.8200	83.1040	155	KX818437	—	—
233.	*P. issikii*	L	ISSIK019-12	N. Kirichenko	*T. amurensis*	Russia	Novosibirsk	54.8200	83.1040	155	KX818432	—	—
234.	*P. issikii*	L	ISSIK018-12	N. Kirichenko	*T. amurensis*	Russia	Novosibirsk	54.8200	83.1040	155	KX818431	—	—

№	Species[1]	Insect stage[2], genitalia slide number	Process ID	Collectors	Host plant[3]	Country	Location[4]	Latitude	Longitude	Elevation	GenBank accession COI	GenBank accession H3	GenBank accession 28S
235.	*P. issikii*	L	ISSIK017-12	N. Kirichenko	*T. amurensis*	Russia	Novosibirsk	54.8200	83.1040	155	KX818430	—	—
236.	*P. issikii*	L	ISSIK020-12	N. Kirichenko	*T. amurensis*	Russia	Novosibirsk	54.8200	83.1040	155	KX818433	KX818861	KX818746
237.	*P. issikii*	L	ISSIK021-12	N. Kirichenko	*T. amurensis*	Russia	Novosibirsk	54.8200	83.1040	155	KX818434	—	—
238.	*P. issikii*	L	ISSIK023-12	N. Kirichenko	*T. sibirica*	Russia	Novosibirsk	54.8200	83.1040	155	KX818436	KX818862	KX818747
239.	*P. issikii*	L	ISSIK022-12	N. Kirichenko	*T. amurensis*	Russia	Novosibirsk	54.8200	83.1040	155	KX818435	—	—
240.	*P. issikii*	L	ISSIK015-12	N. Kirichenko	*T. cordata*	Russia	Novosibirsk	54.8200	83.1040	155	KX818428	—	—
241.	*P. issikii*	A*, 3975♂	ISSIK174-14	N. Kirichenko	*T. mandshurica*	Russia	Ussuriysk, Obs	43.6808	132.1600	224	KX818659	—	—
242.	*P. issikii*	A*, 3983♂	ISSIK162-14	N. Kirichenko	*T. mandshurica*	Russia	Ussuriysk, Obs	43.6808	132.1600	224	KX818656	—	—
243.	*P. issikii*	A*, 3985♂	ISSIK165-14	N. Kirichenko	*T. mandshurica*	Russia	Ussuriysk, Obs	43.6808	132.1600	224	KX818657	—	—
244.	*P. issikii*	A*, 3988♂	ISSIK151-14	N. Kirichenko	*T. mandshurica*	Russia	Ussuriysk, Obs	43.6808	132.1600	224	KX818649	—	—
245.	*P. issikii*	A*, 3990♂	ISSIK170-14	N. Kirichenko	*T. mandshurica*	Russia	Ussuriysk, Obs	43.6808	132.1600	224	KX818658	—	—
246.	*P. issikii*	A*, RFE 20♂	ISSIK152-14	N. Kirichenko	*T. mandshurica*	Russia	Ussuriysk, Obs	43.6808	132.1600	224	KX818650	—	—
247.	*P. issikii*	A*, RFE 21♂	ISSIK155-14	N. Kirichenko	*T. mandshurica*	Russia	Ussuriysk, Obs	43.6808	132.1600	224	KX818652	—	—
248.	*P. issikii*	A*, RFE 22♂	ISSIK181-14	N. Kirichenko	*T. mandshurica*	Russia	Ussuriysk, Obs	43.6808	132.1600	224	KX818664	—	—
249.	*P. issikii*	A*, RFE 23♂	ISSIK180-14	N. Kirichenko	*T. mandshurica*	Russia	Ussuriysk, Obs	43.6808	132.1600	224	KX818663	—	—
250.	*P. issikii*	A*, RFE 24 M♂	ISSIK178-14	N. Kirichenko	*T. mandshurica*	Russia	Ussuriysk, Obs	43.6808	132.1600	224	KX818662	—	—
251.	*P. issikii*	A*, RFE 26 M♂	ISSIK177-14	N. Kirichenko	*T. mandshurica*	Russia	Ussuriysk, Obs	43.6808	132.1600	224	KX818661	—	—
252.	*P. issikii*	A*	ISSIK182-14	N. Kirichenko	*T. mandshurica*	Russia	Ussuriysk, Obs	43.6808	132.1600	224	KX818665	—	—
253.	*P. issikii*	A*	ISSIK183-14	N. Kirichenko	*T. mandshurica*	Russia	Ussuriysk, Obs	43.6808	132.1600	224	KX818666	—	—
254.	*P. issikii*	A*	ISSIK175-14	N. Kirichenko	*T. mandshurica*	Russia	Ussuriysk, Obs	43.6808	132.1600	224	KX818660	—	—
255.	*P. issikii*	A*	ISSIK158-14	N. Kirichenko	*T. mandshurica*	Russia	Ussuriysk, Obs	43.6808	132.1600	224	KX818653	—	—
256.	*P. issikii*	A*	ISSIK154-14	N. Kirichenko	*T. mandshurica*	Russia	Ussuriysk, Obs	43.6808	132.1600	224	KX818651	—	—
257.	*P. issikii*	A	ISSIK159-14	N. Kirichenko	*T. mandshurica*	Russia	Ussuriysk, Obs	43.6808	132.1600	224	KX818654	—	—
258.	*P. issikii*	A	ISSIK161-14	N. Kirichenko	*T. mandshurica*	Russia	Ussuriysk, Obs	43.6808	132.1600	224	KX818655	—	—
259.	*P. issikii*	L	ISSIK384-15	N. Kirichenko	*T. mandshurica*	Russia	Ussuriysk, Obs	43.6808	132.1600	224	KX818545	—	—
260.	*P. issikii*	L	ISSIK379-15	N. Kirichenko	*T. mandshurica*	Russia	Ussuriysk, Obs	43.6810	132.1600	224	KX818448	—	—

№	Species[1]	Insect stage[2], genitalia slide number	Process ID	Collectors	Host plant[3]	Country	Location[4]	Latitude	Longitude	Elevation	GenBank accession COI	GenBank accession H3	GenBank accession 28S
261.	*P. issikii*	L	ISSIK380-15	N. Kirichenko	*T. mandshurica*	Russia	Ussuriysk, Obs	43.6810	132.1600	224	KX818447	—	—
262.	*P. issikii*	L	ISSIK378-15	N. Kirichenko	*T. mandshurica*	Russia	Ussuriysk, Obs	43.6810	132.1600	224	KX818449	—	—
263.	*P. issikii*	L	ISSIK244-14	N. Kirichenko	*T. mandshurica*	Russia	Ussuriysk, Obs	43.6810	132.1600	224	KX818577	—	—
264.	*P. issikii*	L	ISSIK077-14	N. Kirichenko	*T. mandshurica*	Russia	Ussuriysk, Obs	43.6810	132.1600	224	KX818626	—	—
265.	*P. issikii*	L	ISSIK080-14	N. Kirichenko	*T. mandshurica*	Russia	Ussuriysk, Obs	43.6810	132.1600	224	KX818629	—	—
266.	*P. issikii*	L	ISSIK079-14	N. Kirichenko	*T. mandshurica*	Russia	Ussuriysk, Obs	43.6810	132.1600	224	KX818628	—	—
267.	*P. issikii*	L	ISSIK078-14	N. Kirichenko	*T. mandshurica*	Russia	Ussuriysk, Obs	43.6810	132.1600	224	KX818627	—	—
268.	*P. issikii*	L	ISSIK095-14	N. Kirichenko	*T. mandshurica*	Russia	Ussuriysk, Obs	43.6810	132.1600	224	KX818643	—	—
269.	*P. issikii*	L	ISSIK094-14	N. Kirichenko	*T. mandshurica*	Russia	Ussuriysk, Obs	43.6810	132.1600	224	KX818642	—	—
270.	*P. issikii*	L	ISSIK092-14	N. Kirichenko	*T. mandshurica*	Russia	Ussuriysk, Obs	43.6810	132.1600	224	KX818641	—	—
271.	*P. issikii*	L	ISSIK096-14	N. Kirichenko	*T. mandshurica*	Russia	Ussuriysk, Obs	43.6810	132.1600	224	KX818644	—	—
272.	*P. issikii*	A*, RFE 19♂	ISSIK187-14	N. Kirichenko	*T. amurensis*	Russia	Ussuriysk, MTS	43.6886	132.1570	160	KX818668	—	—
273.	*P. issikii*	L	ISSIK033-12	N. Kirichenko	*T. taquetii*	Russia	Ussuriysk, MTS	43.6890	132.1570	160	KX818442	—	—
274.	*P. issikii*	L	ISSIK035-12	N. Kirichenko	*T. taquetii*	Russia	Ussuriysk, MTS	43.6890	132.1570	160	KX818443	KX818863	KX818748
275.	*P. issikii*	L	ISSIK037-12	N. Kirichenko	*T. taquetii*	Russia	Ussuriysk, MTS	43.6890	132.1570	160	KX818444	—	—
276.	*P. issikii*	L	ISSIK039-12	N. Kirichenko	*T. taquetii*	Russia	Ussuriysk, MTS	43.6890	132.1570	160	KX818445	—	—
277.	*P. issikii*	L	ISSIK046-12	N. Kirichenko	*T. mandshurica*	Russia	Ussuriysk, MTS	43.6890	132.1570	160	KX818508	KX818866	KX818751
278.	*P. issikii*	L	ISSIK047-12	N. Kirichenko	*T. mandshurica*	Russia	Ussuriysk, MTS	43.6890	132.1570	160	KX818509	—	—
279.	*P. issikii*	L	ISSIK048-12	N. Kirichenko	*T. mandshurica*	Russia	Ussuriysk, MTS	43.6890	132.1570	160	KX818510	—	—
280.	*P. issikii*	L	ISSIK049-12	N. Kirichenko	*T. mandshurica*	Russia	Ussuriysk, MTS	43.6890	132.1570	160	KX818511	—	—
281.	*P. issikii*	L	ISSIK050-12	N. Kirichenko	*T. mandshurica*	Russia	Ussuriysk, MTS	43.6890	132.1570	160	KX818512	—	—
282.	*P. issikii*	L	ISSIK051-12	N. Kirichenko	*T. mandshurica*	Russia	Ussuriysk, MTS	43.6890	132.1570	160	KX818513	KX818867	KX818752
283.	*P. issikii*	L	ISSIK052-12	N. Kirichenko	*T. mandshurica*	Russia	Ussuriysk, MTS	43.6890	132.1570	160	KX818514	—	—
284.	*P. issikii*	L	ISSIK053-12	N. Kirichenko	*T. mandshurica*	Russia	Ussuriysk, MTS	43.6890	132.1570	160	KX818515	KX818868	KX818753
285.	*P. issikii*	L	ISSIK070-14	N. Kirichenko	*T. mandshurica*	Russia	Ussuriysk, MTS	43.6890	132.1570	160	KX818621	—	—
286.	*P. issikii*	L	ISSIK071-14	N. Kirichenko	*T. mandshurica*	Russia	Ussuriysk, MTS	43.6890	132.1570	160	KX818622	—	—
287.	*P. issikii*	L	ISSIK073-14	N. Kirichenko	*T. mandshurica*	Russia	Ussuriysk, MTS	43.6890	132.1570	160	KX818623	—	—

№	Species[1]	Insect stage[2], genitalia slide number	Process ID	Collectors	Host plant[3]	Country	Location[4]	Latitude	Longitude	Elevation	GenBank accession COI	GenBank accession H3	GenBank accession 28S
288.	*P. issikii*	L	ISSIK074-14	N. Kirichenko	*T. mandshurica*	Russia	Ussuriysk, MTS	43.6890	132.1570	160	KX818624	—	—
289.	*P. issikii*	L	ISSIK076-14	N. Kirichenko	*T. amurensis*	Russia	Ussuriysk, MTS	43.6890	132.1570	160	KX818625	—	—
290.	*P. issikii*	L	ISSIK097-14	N. Kirichenko	*T. mandshurica*	Russia	Ussuriysk, MTS	43.6890	132.1570	160	KX818645	—	—
291.	*P. issikii*	L	ISSIK098-14	N. Kirichenko	*T. mandshurica*	Russia	Ussuriysk, MTS	43.6890	132.1570	160	KX818646	—	—
292.	*P. issikii*	L	ISSIK099-14	N. Kirichenko	*T. taquetii*	Russia	Ussuriysk, MTS	43.6890	132.1570	160	KX818647	—	—
293.	*P. issikii*	L	ISSIK101-14	N. Kirichenko	*T. taquetii*	Russia	Ussuriysk, MTS	43.6890	132.1570	160	KX818648	—	—
294.	*P. issikii*	L	ISSIK186-14	N. Kirichenko	*T. amurensis*	Russia	Ussuriysk, MTS	43.6886	132.1570	160	KX818667	—	—
295.	*P. issikii*	L	ISSIK242-14	N. Kirichenko	*T. mandshurica*	Russia	Ussuriysk, MTS	43.6890	132.1570	160	KX818576	—	—
296.	*P. issikii*	L	ISSIK268-14	N. Kirichenko	*T. mandshurica*	Russia	Ussuriysk, MTS	43.6890	132.1570	160	KX818600	—	—
297.	*P. issikii*	L	ISSIK269-14	N. Kirichenko	*T. taquetii*	Russia	Ussuriysk, MTS	43.6890	132.1570	160	KX818601	—	—
298.	*P. issikii*	L	ISSIK381-15	N. Kirichenko	*T. mandshurica*	Russia	Ussuriysk, MTS	43.6886	132.1570	160	KX818507	—	—
299.	*P. issikii*	L	ISSIK383-15	N. Kirichenko	*T. mandshurica*	Russia	Ussuriysk, MTS	43.6890	132.1570	160	KX818546	—	—
300.	*P. issikii*	L	ISSIK081-14	V. Ponomarev	*T. cordata*	Russia	Yekaterinburg	56.8170	60.4490	300	KX818630	—	—
301.	*P. issikii*	L	ISSIK245-14	V. Ponomarev	*T. cordata*	Russia	Yekaterinburg	56.8170	60.4490	300	KX818578	—	—
302.	*P. issikii*	L	ISSIK246-14	V. Ponomarev	*T. cordata*	Russia	Yekaterinburg	56.8170	60.4490	300	KX818579	—	—
303.	*P. issikii*	L	ISSIK247-14	V. Ponomarev	*T. cordata*	Russia	Yekaterinburg	56.8170	60.4490	300	KX818580	—	—
304.	*P. issikii*	L	ISSIK248-14	V. Ponomarev	*T. cordata*	Russia	Yekaterinburg	56.8170	60.4490	300	KX818581	—	—
305.	*P. issikii*	L	ISSIK359-14	V. Ponomarev	*T. cordata*	Russia	Yekaterinburg	56.8170	60.4490	300	KX818553	—	—
306.	*P. issikii*	L	ISSIK360-14	V. Ponomarev	*T. cordata*	Russia	Yekaterinburg	56.8170	60.4490	300	KX818552	—	—
307.	*P. issikii*	L	ISSIK064-14	N. Kirichenko	*T. cordata*	Russia	Tyumen	57.1470	65.5250	82	KX818616	—	—
308.	*P. issikii*	L	ISSIK065-14	N. Kirichenko	*T. cordata*	Russia	Tyumen	57.1470	65.5250	82	KX818617	—	—
309.	*P. issikii*	L	ISSIK066-14	N. Kirichenko	*T. cordata*	Russia	Tyumen	57.1470	65.5250	82	KX818618	—	—
310.	*P. issikii*	L	ISSIK067-14	N. Kirichenko	*T. cordata*	Russia	Tyumen	57.1470	65.5250	82	KX818619	—	—
311.	*P. issikii*	L	ISSIK068-14	N. Kirichenko	*T. cordata*	Russia	Tyumen	57.1470	65.5250	82	KX818620	—	—
312.	*P. issikii*	L	ISSIK357-14	N. Kirichenko	*T. cordata*	Russia	Tyumen	57.1470	65.5250	82	KX818555	—	—
313.	*P. issikii*	L	ISSIK358-14	N. Kirichenko	*T. cordata*	Russia	Tyumen	57.1470	65.5250	82	KX818554	—	—
314.	*P. issikii*	L	ISSIK270-14	V. Zolotuhin	*T. cordata*	Russia	Ulyanovsk	54.3120	48.3800	132	KX818602	—	—
315.	*P. issikii*	L	ISSIK271-14	V. Zolotuhin	*T. cordata*	Russia	Ulyanovsk	54.3120	48.3800	132	KX818603	—	—

№	Species[1]	Insect stage[2], genitalia slide number	Process ID	Collectors	Host plant[3]	Country	Location[4]	Latitude	Longitude	Elevation	GenBank accession COI	GenBank accession H3	GenBank accession 28S
316.	*P. issikii*	L	ISSIK272-14	V .Zolotuhin	*T. cordata*	Russia	Ulyanovsk	54.3120	48.3800	132	KX818604	—	—
317.	*P. issikii*	L	ISSIK273-14	V.Zolotuhin	*T. cordata*	Russia	Ulyanovsk	54.3120	48.3800	132	KX818605	—	—
318.	*P. issikii*	L	ISSIK354-14	V. Zolotuhin	*T. cordata*	Russia	Ulyanovsk	54.3120	48.3800	132	KX818558	—	—
319.	*P. issikii*	L	ISSIK355-14	V. Zolotuhin	*T. cordata*	Russia	Ulyanovsk	54.3120	48.3800	132	KX818557	—	—
320.	*P. issikii*	L	ISSIK356-14	V. Zolotuhin	*T. cordata*	Russia	Ulyanovsk	54.3120	48.3800	132	KX818556	—	—
321.	*P. issikii*	A*, SK 1♂	MICRU020-15	B.-K. Byun	*T. mandshurica*	S. Korea	Boeun-gun	36.3870	127.8810	274	KX818523	KX818869	KX818754
322.	*P. issikii*	A*, SK 2♂	MICRU022-15	B.-K. Byun	*T. mandshurica*	S. Korea	Boeun-gun	36.3870	127.8810	274	KX818521	KX818521	—
323.	*P. issikii*	A*	MICRU021-15	B.-K. Byun	*T. mandshurica*	S. Korea	Boeun-gun	36.3870	127.8810	274	KX818522	KX818522	—
324.	*P. issikii*	A*	MICRU023-15	B.-K. Byun	*T. mandshurica*	S. Korea	Boeun-gun	36.3870	127.8810	274	KX818520	KX818520	—
325.	*P. issikii*	L	MICRU024-15	B.-K. Byun	*T. mandshurica*	S. Korea	Boeun-gun	36.3870	127.8810	274	KX818519	KX818519	—
326.	*P. issikii*	A	GRSLO518-11	S. Gomboc	*Tilia sp.*	Slovenia	Ljubljanska kotlina	46.2370	14.3360	423	JN280342	—	—
327.	*P. issikii*	A	GRSLO597-11	S. Gomboc	—	Slovenia	Kranj	46.2380	14.3460	380	—	JN299411	—
328.	*P. issikii*	A	ISSIK318-14	S. Gomboc	*T. cordata*	Slovenia	Kranj	46.2360	14.3320	407	KX818396	KX818396	—
329.	*P. issikii*	A	ISSIK319-14	S. Gomboc	*T. cordata*	Slovenia	Kranj	46.2360	14.3320	407	KX818395	KX818395	—
330.	*P. issikii*	L	ISSIK320-14	S. Gomboc	*T. cordata*	Slovenia	Kranj	46.2360	14.3320	407	KX818464	KX818464	—
331.	*P. issikii*	L	ISSIK321-14	S. Gomboc	*T. cordata*	Slovenia	Kranj	46.2360	14.3320	407	KX818463	KX818463	—
332.	*P. issikii*	L	ISSIK275-14	M. Tomoshevich	*T. cordata*	Ukraine	Kiev	50.4150	30.5620	168	KX818491	KX818491	—
333.	*P. issikii*	L	ISSIK276-14	M. Tomoshevich	*T. cordata*	Ukraine	Kiev	50.4150	30.5620	168	KX818492	KX818492	—
334.	*P. issikii*	L	ISSIK277-14	M. Tomoshevich	*T. cordata*	Ukraine	Kiev	50.4150	30.5620	168	KX818493	KX818493	—
335.	*P. issikii*	P	ISSIK278-14	M. Tomoshevich	*T. cordata*	Ukraine	Kiev	50.4150	30.5620	168	KX818494	KX818494	—
336.	*P. issikii*	L	ISSIK279-14	M. Tomoshevich	*T. cordata*	Ukraine	Kiev	50.4150	30.5620	168	KX818495	KX818495	—
337.	*P. issikii*	L	ISSIK280-14	M. Tomoshevich	*T. cordata*	Ukraine	Kiev	50.4150	30.5620	168	KX818496	KX818496	—
338.	*P. issikii*	L	ISSIK281-14	M. Tomoshevich	*T. cordata*	Ukraine	Kiev	50.4150	30.5620	168	KX818497	KX818497	—
339.	*P. issikii*	L	ISSIK348-14	M. Tomoshevich	*T. cordata*	Ukraine	Kiev	50.4150	30.5620	168	KX818564	KX818564	—
340.	*P. issikii*	L	ISSIK349-14	M. Tomoshevich	*T. cordata*	Ukraine	Kiev	50.4150	30.5620	168	KX818563	KX818563	—
341.	*P. issikii*	L	ISSIK350-14	M. Tomoshevich	*T. cordata*	Ukraine	Kiev	50.4150	30.5620	168	KX818562	KX818562	—
342.	*P. issikii*	L	ISSIK351-14	M. Tomoshevich	*T. cordata*	Ukraine	Kiev	50.4150	30.5620	168	KX818561	KX818561	—
343.	*P. issikii*	L	ISSIK352-14	M. Tomoshevich	*T. cordata*	Ukraine	Kiev	50.4150	30.5620	168	KX818560	KX818560	—

№	Species[1]	Insect stage[2], genitalia slide number	Process ID	Collectors	Host plant[3]	Country	Location[4]	Latitude	Longitude	Elevation	GenBank accession COI	GenBank accession H3	GenBank accession 28S
344.	*P. issikii*	L	ISSIK353-14	M. Tomoshevich	*T. cordata*	Ukraine	Kiev	50.4150	30.5620	168	KX818559	KX818559	—
345.	*Phyllonorycter* sp. n	A*, Sendai 14♂	ISSIK307-14	I. Ohshima	*T. japonica*	Japan	Sendai	35.0100	135.7980	244	KX818706	KX818878	KX818764
346.	*P.* sp. n	A*, Sendai 15♂	ISSIK308-14	I. Ohshima	*T. japonica*	Japan	Sendai	35.0100	135.7980	244	KX818727	KX818892	KX818778
347.	*P.* sp. n	A*	ISSIK309-14	I. Ohshima	*T. japonica*	Japan	Sendai	35.0100	135.7980	244	KX818729	KX818894	KX818780
348.	*P.* sp. n	A*	ISSIK325-14	I. Ohshima	*T. japonica*	Japan	Sendai	35.0100	135.7980	244	KX818705	KX818877	KX818763
349.	*P.* sp. n	A*	ISSIK326-14	I. Ohshima	*T. japonica*	Japan	Sendai	35.0100	135.7980	244	KX818708	KX818879	KX818765
350.	*P.* sp. n	A	ISSIK372-14	T. Kanbe	*T. japonica*	Japan	Sendai	35.0100	135.7980	244	KX818697	KX818697	—
351.	*P.* sp. n	A	ISSIK373-14	T. Kanbe	*T. japonica*	Japan	Sendai	35.0100	135.7980	244	KX818696	KX818696	—
352.	*P.* sp. n	L	ISSIK283-14	I. Ohshima	*T. japonica*	Japan	Sendai	35.0100	135.7980	244	KX818719	KX818887	KX818773
353.	*P.* sp. n	L	ISSIK284-14	I. Ohshima	*T. japonica*	Japan	Sendai	35.0100	135.7980	244	KX818693	KX818875	KX818760
354.	*P.* sp. n	L	ISSIK286-14	I. Ohshima	*T. japonica*	Japan	Sendai	35.0100	135.7980	244	KX818712	KX818882	KX818768
355.	*P.* sp. n	L	ISSIK285-14	I. Ohshima	*T. japonica*	Japan	Sendai	35.0100	135.7980	244	KX818709	KX818880	KX818766
356.	*P.* sp. n	L	ISSIK370-14	T. Kanbe	*T. japonica*	Japan	Sendai	35.0100	135.7980	244	KX818699	KX818699	—
357.	*P.* sp. n	L	ISSIK371-14	T. Kanbe	*T. japonica*	Japan	Sendai	35.0100	135.7980	244	KX818698	KX818698	—
358.	*P.* sp. n	L	ISSIK369-14	T. Kanbe	*T. japonica*	Japan	Sendai	35.0100	135.7980	244	KX818702	KX818702	—
359.	*P.* sp. n	L	ISSIK368-14	T. Kanbe	*T. japonica*	Japan	Sendai	35.0100	135.7980	244	KX818703	KX818703	—
360.	*P.* sp. n	P	ISSIK374-14	T. Kanbe	*T. japonica*	Japan	Sendai	35.0100	135.7980	244	KX818695	KX818695	—
361.	*P.* sp. n	A	WOGRA073-15	C. Doorenweerd	*Tilia sp*	Japan	Kuroishi	40.6410	140.7532	54	KY079348	—	—
362.	*P.* sp. n	A	ISSIK299-14	T. Kanbe	*T. maximowicziana*	Japan	Sapporo	43.0340	141.3150	121	KX818722	KX818889	KX818775
363.	*P.* sp. n	A	ISSIK305-14	T. Kanbe	*T. maximowicziana*	Japan	Sapporo	43.0340	141.3150	121	KX818726	KX818891	KX818777
364.	*P.* sp. n	L	ISSIK306-14	T. Kanbe	*T. maximowicziana*	Japan	Sapporo	43.0340	141.3150	121	KX818690	KX818873	KX818758
365.	*P.* sp. n	L	ISSIK034-12	N. Kirichenko	*T. taquetii*	Russia	Ussuriysk, MTS	43.6890	132.1570	160	KX818713	KX818883	KX818769
366.	*P.* sp. n	L	ISSIK036-12	N. Kirichenko	*T. taquetii*	Russia	Ussuriysk, MTS	43.6890	132.1570	160	KX818711	KX818711	—
367.	*P.* sp. n	L	ISSIK038-12	N. Kirichenko	*T. taquetii*	Russia	Ussuriysk, MTS	43.6890	132.1570	160	KX818710	KX818881	KX818767
368.	*P.* sp. n	L	ISSIK040-12	N. Kirichenko	*T. taquetii*	Russia	Ussuriysk, MTS	43.6890	132.1570	160	KX818707	KX818707	—
369.	*P.* sp. n	L	ISSIK041-12	N. Kirichenko	*T. amurensis*	Russia	Ussuriysk, MTS	43.6890	132.1570	160	KX818704	KX818876	KX818762
370.	*P.* sp. n	L	ISSIK042-12	N. Kirichenko	*T. amurensis*	Russia	Ussuriysk, MTS	43.6890	132.1570	160	KX818694	KX818694	—

№	Species[1]	Insect stage[2], genitalia slide number	Process ID	Collectors	Host plant[3]	Country	Location[4]	Latitude	Longitude	Elevation	GenBank accession COI	GenBank accession H3	GenBank accession 28S
371.	*P.* sp. n	L	ISSIK043-12	N. Kirichenko	*T. amurensis*	Russia	Ussuriysk, MTS	43.6890	132.1570	160	KX818689	KX818689	—
372.	*P.* sp. n	L	ISSIK044-12	N. Kirichenko	*T. amurensis*	Russia	Ussuriysk, MTS	43.6890	132.1570	160	KX818728	KX818893	KX818779
373.	*P.* sp. n	L	ISSIK045-12	N. Kirichenko	*T. amurensis*	Russia	Ussuriysk, MTS	43.6890	132.1570	160	KX818688	KX818872	KX818757
374.	*P.* sp. n	L	ISSIK072-14	N. Kirichenko	*T. mandshurica*	Russia	Ussuriysk, MTS	43.6890	132.1570	160	KX818721	KX818721	—
375.	*P.* sp. n	L	ISSIK075-14	N. Kirichenko	*T. amurensis*	Russia	Ussuriysk, MTS	43.6890	132.1570	160	KX818724	KX818724	—
376.	*P.* sp. n	L	ISSIK100-14	N. Kirichenko	*T. taquetii*	Russia	Ussuriysk, MTS	43.6890	132.1570	160	KX818723	KX818723	—
377.	*P.* sp. n	L	ISSIK241-14	N. Kirichenko	*T. mandshurica*	Russia	Ussuriysk, MTS	43.6890	132.1570	160	KX818701	KX818701	—
378.	*P.* sp. n	L	ISSIK382-15	N. Kirichenko	*T. mandshurica*	Russia	Ussuriysk, MTS	43.6886	132.1570	160	KX818691	KX818691	—
379.	*P.* sp. n	P	ISSIK243-14	N. Kirichenko	*T. amurensis*	Russia	Ussuriysk, MTS	43.6890	132.1570	160	KX818692	KX818874	KX818759
380.	*P.* sp. n	L	ISSIK093-14	N. Kirichenko	*T. mandshurica*	Russia	Ussuriysk, Obs	43.6810	132.1600	224	KX818714	KX818714	—
381.	*P.* sp. n	P	ISSIK267-14	N. Kirichenko	*T. mandshurica*	Russia	Ussuriysk, Obs	43.6810	132.1600	224	KX818725	KX818890	KX818776
382.	*P.* sp. n	L	ISSIK029-12	N. Kirichenko	*Tilia sp.*	Russia	Sikhote-Alin Mts, CT	43.5990	134.3000	690	KX818720	KX818888	KX818774
383.	*P.* sp. n	L	ISSIK030-12	N. Kirichenko	*Tilia sp.*	Russia	Sikhote-Alin Mts, CT	43.5990	134.3000	690	KX818717	KX818886	KX818772
384.	*P.* sp. n	L	ISSIK031-12	N. Kirichenko	*Tilia sp.*	Russia	Sikhote-Alin Mts, CT	43.5990	134.3000	690	KX818716	KX818885	KX818771
385.	*P.* sp. n	L	ISSIK032-12	N. Kirichenko	*Tilia sp.*	Russia	Sikhote-Alin Mts, CT	43.5990	134.3000	690	KX818715	KX818884	KX818770
386.	*P.* sp. n	L	ISSIK240-14	N. Kirichenko	*T. mandshurica*	Russia	Vladivostok	43.1940	131.9210	82	KX818700	KX818700	—
387.	*P.* sp. n	P	ISSIK069-14	N. Kirichenko	*T. mandshurica*	Russia	Vladivostok	43.1940	131.9210	82	KX818718	KX818718	—
**Outrgoup**	*Phyllonorycter lucetiella*	P	MICRU060-15	J-.F. Landry	*T. americana*	Canada	Quebec	45.4690	-75.8118	54	KX818687	KX818871	KX818756

[1]Species: *Phyllonorycter issikii* (Kumata, 1963), *Phyllonorycter* sp. n (a putative new cryptic species), *Phyllonorycter lucetiella* (Clemens, 1859) (outgroup); [2]Insect stage: L – larva, P – pupa, A – adult; [3]Host plant: – no data; [4]Location: in the Russian Far East (MTS – arboretum of the Mountain-taiga station of the Far Eastern branch of the Russian Academy of Sciences; Obs – Astrophysical observatory; CT – the national park “Call of the Tiger” in the Sikhote-Alin mountains), where either of the two species was present.

---

[i] Overall, 387 specimens were involved in the study: 377 specimens of *Phyllonorycter issikii* and the putative new species were studied genetically, 72 specimens were studied morphologically (62 out of 72 specimens were DNA-barcoded). Adults, which wing pattern was examined are marked by *, the dissected specimens are supplied with genitalia slide number (see column: Insect stage, genitalia slide number). Additionally one specimen of *Phyllonorycter lucetiella* was used as an outgroup to root the genetic trees.
