# Supplementary material for: From east to west across the Palearctic: Phylogeography of the invasive lime leaf miner Phyllonorycter issikii (Lepidoptera: Gracillariidae) and discovery of a putative new cryptic species in East Asia
Source: PLoS One. 2017 Feb 10;12(2):e0171104. doi: 10.1371/journal.pone.0171104 (PMC5302804; doi:10.1371/journal.pone.0171104)
Supplement: S2 Table — This pdf file contains the data included in this manuscript. (PDF) [file pone.0171104.s003.pdf]

Supplementary material

S2 Table. Presence of *Phyllonorycter issikii* haplotypes in different countries in the Palearctic.

Haplotype	Country[1]
H1	AT, BG, CZ, FI, DE, JP, HU, NL, PL, RU (West), SI, UA
H2	RU (Sib)
H3	DE, RU (Sib)
H4	JP
H5	AT
H6	LT
H7	FI
H8	AT, BG, FI, DE, HU, IT , LT, RU (West), RU (Sib), RU (RFE), SI, UA
H9	RU (West, Sib)
H10	FI
H11	PL, RU (West)
H12	FI
H13	RU (Sib)
H14	AT, FI, HU, RU (Sib)
H15	UA
H16	RU (West)
H17	AT, BG, FI, IT, LT, RU (West), RU (Sib), SI, UA,
H18	AT
H19	FI, RU (Sib)
H20	HU
H21	RU (West)
H22	RU (West)
H23	AT, BG, FI, DE, HU, LT, NL, PL, RU (West), RU (Sib), UA
H24	AT, RU (West)
H25	RU (RFE)
H26	RU (RFE)
H27	CH
H28	RU (RFE)
H28	SK
H30	RU (RFE)
H31	RU (RFE)

[1]**Countries:** AT – Austria, BG – Bulgaria, CH – China, CZ – Czech Republic, DE – Germany, FI – Finland, HU – Hungary, IT – Italy, JP – Japan, LT – Lithuania, NL – Netherlands, PL – Poland, SI – Slovenia, SK – South Korea, UA – Ukraine.

RU – Russia, due to the large size of the country, is mentioned by regions: RU (West) – Western Russia, RU(Sib) – Siberia and RU(RFE) – Russian Far East.

The countries shaded in red represent the putative native range of *Phyllonorycter issikii*, the others represent its putative invasive range.
