# Supplementary material for: From east to west across the Palearctic: Phylogeography of the invasive lime leaf miner Phyllonorycter issikii (Lepidoptera: Gracillariidae) and discovery of a putative new cryptic species in East Asia
Source: PLoS One. 2017 Feb 10;12(2):e0171104. doi: 10.1371/journal.pone.0171104 (PMC5302804; doi:10.1371/journal.pone.0171104)
Supplement: S3 Table — This pdf file contains the data included in this manuscript. (PDF) [file pone.0171104.s004.pdf]

Supplementary material

S3 Table. Distribution of COI sequence divergences (K2P) at *Phyllonorycter issikii* and the putative *Phyllonorycter* sp. n.

Taxonomic level	n	Taxa	Comparisons	Genetic distance, (%)		
				Min	Mean ± StDiv	Max
Intraspecific	377	2	56514	0	1.27±0.04	2.96
Interspecific	377	1	14362	3.66	5.13±0.003	6.02
